# From lithium to sodium: cell chemistry of room temperature sodium–air and sodium–sulfur batteries

**DOI:** 10.3762/bjnano.6.105

**Published:** 2015-04-23

**Authors:** Philipp Adelhelm, Pascal Hartmann, Conrad L Bender, Martin Busche, Christine Eufinger, Juergen Janek

**Affiliations:** 1 Institute for Technical Chemistry and Environmental Chemistry, Center for Energy and Environmental Chemistry, Friedrich-Schiller-University Jena, Lessingstraße 12, 07743 Jena, Germany; 2 Institute of Physical Chemistry, Justus-Liebig-University Giessen, Heinrich-Buff-Ring 58, 35392 Giessen, Germany; 3 BASF SE, 67056 Ludwigshafen, Germany; 4 Battery and Electrochemistry Laboratory, Institute of Nanotechnology, Karlsruhe Institute of Technology, Hermann-von-Helmholtz-Platz 1, 76344 Eggenstein-Leopoldshafen, Germany

**Keywords:** energy storage, lithium–oxygen battery, lithium–sulfur battery, sodium–oxygen battery, sodium–sulfur battery

## Abstract

Research devoted to room temperature lithium–sulfur (Li/S_8_) and lithium–oxygen (Li/O_2_) batteries has significantly increased over the past ten years. The race to develop such cell systems is mainly motivated by the very high theoretical energy density and the abundance of sulfur and oxygen. The cell chemistry, however, is complex, and progress toward practical device development remains hampered by some fundamental key issues, which are currently being tackled by numerous approaches. Quite surprisingly, not much is known about the analogous sodium-based battery systems, although the already commercialized, high-temperature Na/S_8_ and Na/NiCl_2_ batteries suggest that a rechargeable battery based on sodium is feasible on a large scale. Moreover, the natural abundance of sodium is an attractive benefit for the development of batteries based on low cost components. This review provides a summary of the state-of-the-art knowledge on lithium–sulfur and lithium–oxygen batteries and a direct comparison with the analogous sodium systems. The general properties, major benefits and challenges, recent strategies for performance improvements and general guidelines for further development are summarized and critically discussed. In general, the substitution of lithium for sodium has a strong impact on the overall properties of the cell reaction and differences in ion transport, phase stability, electrode potential, energy density, etc. can be thus expected. Whether these differences will benefit a more reversible cell chemistry is still an open question, but some of the first reports on room temperature Na/S_8_ and Na/O_2_ cells already show some exciting differences as compared to the established Li/S_8_ and Li/O_2_ systems.

## Review

### 1 Introduction

**Figure 1:**
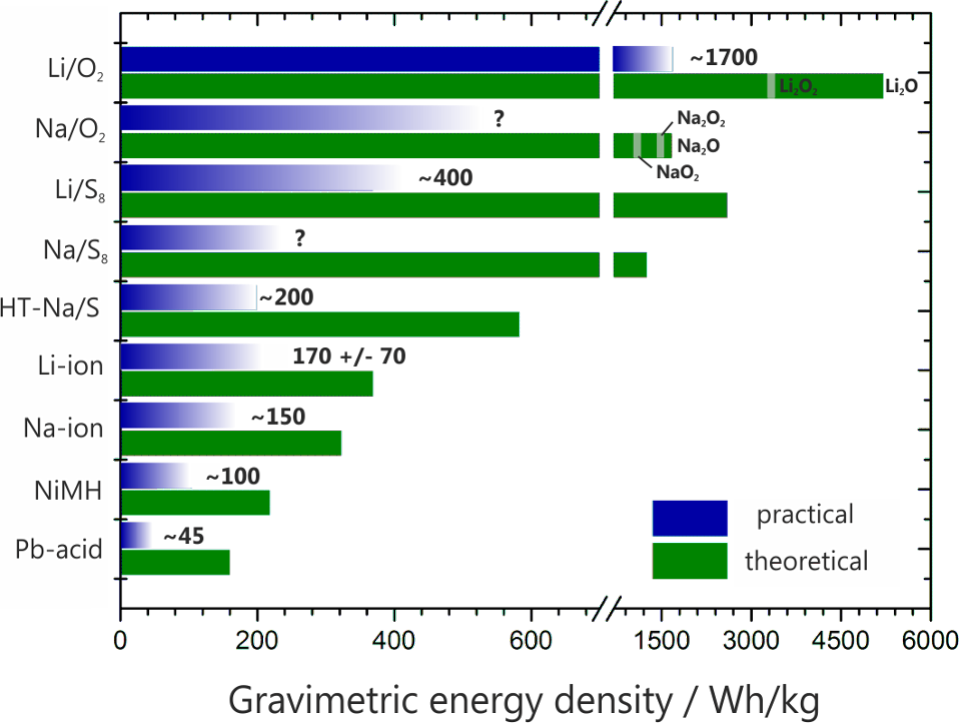
Theoretical and (estimated) practical energy densities of different rechargeable batteries: Pb–acid – lead acid, NiMH – nickel metal hydride, Na-ion – estimate derived from data for Li-ion assuming a slightly lower cell voltage, Li-ion – average over different types, HT-Na/S_8_ – high temperature sodium–sulfur battery, Li/S_8_ and Na/S_8_ – lithium–sulfur and sodium–sulfur battery assuming Li_2_S and Na_2_S as discharge products, Li/O_2_ and Na/O_2_ – lithium–oxygen battery (theoretical values include the weight of oxygen and depend on the stoichiometry of the assumed discharge product, i.e., oxide, peroxide or superoxide). Note that the values for practical energy densities can largely vary depending on the battery design (size, high power, high energy, single cell or battery) and the state of development. All values for practical energy densities refer to the cell level (except Pb–acid, 12 V). The values for the Li/S_8_ and Li/O_2_ batteries were taken from the literature (cited within the main text) and are used to estimate the energy densities for the Na/S_8_ and Na/O_2_ cells. Of the above technologies, only the lead acid, NiMH, Li-ion and high temperature Na/S_8_ technologies have been commercialized to date.

Rechargeable lithium-ion batteries (LIBs) have rapidly become the most important form of energy storage for all mobile applications since their commercialization in the early 1990s. This is mainly due to their unrivaled energy density that easily surpasses other rechargeable battery systems such as metal–hydride or lead–acid. However, the ongoing need to store electricity even more safely, more compactly and more affordably necessitates continuous research and development. The need for inexpensive stationary energy storage has become an additional challenge, which also triggers research on alternative batteries. Major efforts are directed towards continuous improvements of the different Li-ion technologies by more efficient packaging, processing, better electrolytes and optimized electrode materials, for example. Although significant progress has been achieved with respect to the power density over the last years, the increase in energy density (volumetrically and gravimetrically) was relatively small [1]. A comparison of different battery technologies with respect to their energy densities is shown in Figure 1.

**Figure 2:**
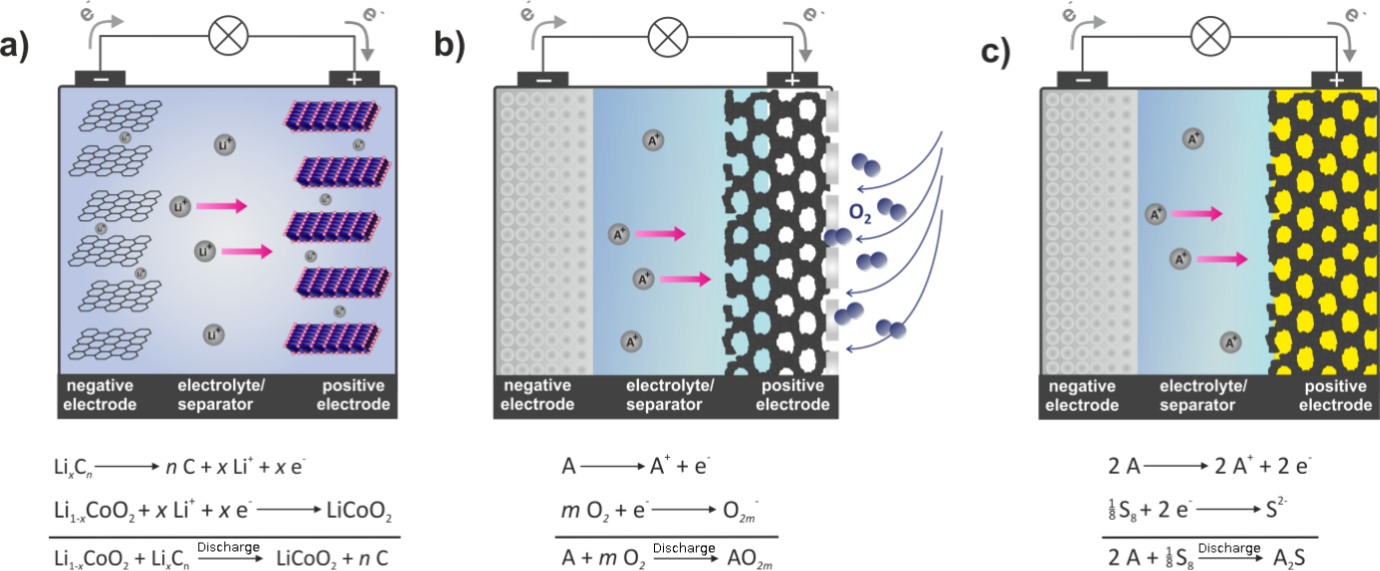
Operating principles of (a) a lithium-ion battery, (b) a metal–oxygen battery (non-aqueous electrolyte) and (c) a metal–sulfur battery during discharge. (A = Li, Na). A lithium-ion battery is based on intercalation compounds as electrodes. The exact cell reaction depends on the materials used. In this example, the reaction equation is formulated for the classical LIB with graphite as the negative and LiCoO_2_ as the positive electrode. The same concept can be applied for a sodium-ion battery. Metal–oxygen and metal–sulfur batteries perform best with a lithium or sodium metal as the anode. The positive electrode consists of a porous support, usually carbon. In a metal–oxygen battery, this support enables the reduction of atmospheric oxygen and accommodates the insulating discharge products of Li_2_O_2_, Na_2_O_2_, NaO_2_, or ideally, Li_2_O and Na_2_O. In metal–sulfur batteries, the support hosts the insulating end members of the cell reaction, which are sulfur (before discharge) and ideally Li_2_S and Na_2_S (after discharge). The sketch in Figure 2 illustrates the most frequently studied cell concepts for metal–oxygen and metal–sulfur cells. Other concepts, for example, solid electrolytes or liquid electrodes, are also currently being studied.

Ultimately, the energy density of a practical battery is determined by the cell reaction itself, that is, the electrode materials being used. The need for a proper cell design and packaging considerably reduces the practical energy density of a battery compared to the theoretical energy density. The cell reaction of Li-ion batteries is not fixed and different electrode materials and mixtures are used depending on the type of application. Graphite/carbon and to a lesser degree Li_4/3_Ti_5/3_O_4_ (LTO) serve as the negative electrodes. Recently, silicon has been added in small amounts to graphite to increase the capacity. Layered oxides (the classic LiCoO_2_, LCO) and related materials (LiNi_1−_*_x−y_*Mn*_x_*Co*_y_*O_2_, NMC; LiNi_0.8_Co_0.15_Al_0.05_O_2_, NCA; olivines, LiFePO_4_, LFP; spinels, LiMn_2_O_4_, LMO) are applied as positive electrodes. The underlying storage principle of all these electrode materials is a one-electron transfer per formula unit. In this process, the de-/intercalation of one Li-ion is linked to a change in the transition metal oxidation state by one (Co^3+/4+^, Fe^2+/3+^, Mn^3+/4+^, etc.), as illustrated in Figure 2a. However, since the positive electrode materials often suffer from stability issues at too low lithium contents, only a fraction of the theoretical capacity can be achieved in practice (with LFP being an exception). For example, only 0.5 electrons per formula unit can be reversibly exchanged for LCO. The electrode reaction for LCO can therefore be written as

[1]
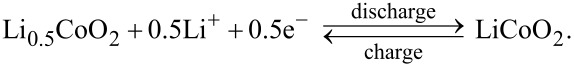


The amount of charge that can be stored during this process is therefore limited and the capacities of positive insertion-type and intercalation-type electrode materials are around 120–180 mAh/g. Employing graphite as a negative electrode (372 mAh/g), the theoretical energy densities of single cells for current Li-ion technology are limited to around 350–400 Wh/kg and 1200–1400 Wh/L. Roughly about one fourth to one half is achieved in practice due to the additional weight and volume of the current collectors, separator, electrolyte, cell housing, and so forth.

Significantly higher energy densities can only be achieved by using electrode reactions such as multielectron transfer and/or lighter elements. A broad range of so-called conversion reactions has been studied which are based on the full reduction of the transition metal [2]. The general electrode reaction can be written as:

[2]
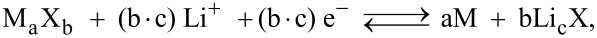


where M is either a transition metal (Cu, Co, Fe, etc.) or Mg, and X is an anion (F, O, S, etc.). The overall success has been limited as conversion reactions typically show large irreversible capacities during the first cycle and a large hysteresis during cycling. This irreversible capacity is mostly caused by the need for complete lattice reconstruction and the corresponding formation of new interfaces.

The most appealing multielectron transfer systems are the lithium–sulfur battery and the lithium–air (or more precisely, the lithium–oxygen battery) in which a non-metal is the redox-active element. Both batteries combine very high theoretical energy densities with the advantage of using abundant and thus resource-uncritical elements. Both systems have been intensively studied over the last years. For example, more than 250 publications appeared in the field of lithium–sulfur batteries in 2014 alone and about 200 publications in 2014 are concerned with lithium–oxygen batteries. The cell concepts are entirely different from conventional Li-ion technology, as depicted in Figure 2. Here, elemental sulfur and atmospheric oxygen are reduced at the positive electrode to form Li_2_S and Li_2_O_2_ during discharge, which is expressed by:

[3]
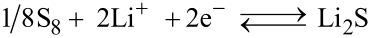


[4]
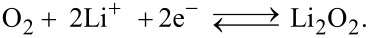


Moreover, the cells ideally operate with metallic lithium as the negative electrode. No heavy transition metals participate in the cell reaction and theoretical energy densities of 2613 Wh/kg for the Li/S_8_ and 3458 Wh/kg for the Li/O_2_ cell can be calculated.

Perhaps the most important conceptual differences between these cell systems and Li-ion batteries are (1) that the redox centers (oxygen and sulfur) are lighter and spatially more concentrated, allowing for higher energy densities and (2) that the redox-active (molecular) species are mobile in liquid electrolytes and new phases form and decompose during cycling. In intercalation compounds, the redox centers (transition metal cations) are immobile as they are pinned to the fixed positions of the crystal lattice and are, therefore, spatially diluted. However, due to the poor conductivity of sulfur, Li_2_S and Li_2_O_2_, the non-metal redox materials also require a suitable conductive support structure. For the Li/S_8_ and Li/O_2_ batteries, this means that significant complexity is added, as a series of transport steps and nucleation/decomposition processes take place that will depend on the morphology, microstructure and surface chemistry of the conductive support. Side reactions with the metallic anode and dendrite formation further complicate the cell chemistry, and therefore, the cycle life of both cell systems remains insufficient to date. The Li/O_2_ cell particularly suffers from additional side reactions related to electrolyte decomposition at the positive electrode. Many challenges therefore must be tackled in order to develop practical systems.

Research on sodium-ion batteries (NIBs) has recently been revived and is largely motivated by the natural abundance of sodium [3–10]. The sodium content in the earth’s crust and water amount to 28,400 mg/kg and 11,000 mg/L compared to 20 mg/kg and 0.18 mg/L for lithium [11]. Additionally, the number of known sodium compounds is much larger as compared to lithium, and thus combinations of electrode materials that enable the development of batteries based solely on low cost elements (or that provide specific advantages that complement Li-ion technology in special applications) are expected. It is interesting to note that sodium-ion and lithium-ion batteries were studied in the 1970s and 1980s. However, due to the success of the lithium-ion battery (and probably the insufficient overall quality of materials, electrolytes and glove boxes [3]), research on sodium-based batteries was largely abandoned. The only exceptions were the high temperature systems Na/S_8_ and Na/NiCl_2_ [12–15].

Although one would initially assume very similar cell chemistries for otherwise identical LIBs and NIBs, the behavior is in most cases quite different. The reason is related to the larger size of the sodium ion that affects the phase stability, the transport properties and the interphase formation. The basic characteristics of multielectron transfer reactions involving sodium-based conversion reactions have been recently summarized and appear quite attractive. However, similar challenges compared to lithium-based conversion reactions are also found [10].

The intriguing question is whether the chemical differences between sodium and lithium could help to solve some of the challenges known for the Li/S_8_ and Li/O_2_ cells. Although an unavoidable penalty with respect to the energy density is paid when replacing lithium by sodium, the theoretical value for a room-temperature Na/S_8_ battery with Na_2_S as a discharge product (1273 Wh/kg) and a Na/O_2_ cell with Na_2_O_2_ as a discharge product (1600 Wh/kg) are still very high compared to LIBs. However, to date, only very little is known about the room temperature chemistry of Na/S_8_ and Na/O_2_ cells. Only around thirty studies have been published as of 2014 in total. Although there is some dispute about the stoichiometry of the discharge products in these cells, it has been demonstrated that Na/O_2_ cells can be cycled with much better performance as compared to the analogue Li/O_2_ cell. Replacing lithium by sodium might therefore be an effective strategy to improve the reversibility of high energy battery systems, notwithstanding the reduced theoretical energy capacity.

Some general differences between lithium and sodium cells are immediately apparent:

1. The lower melting point of sodium (*T*_m,Na_ = 98 °C) as compared to lithium (*T*_m,Li_ = 181 °C) and its generally higher chemical reactivity pose additional safety issues for cells using metal anodes. On the other hand, cell concepts with a molten anode might be easier to realize given the advantages of better kinetics and prevention of dendrite formation.
2. Sodium is softer than lithium, making handling and processing more difficult. On the other hand, avoiding dendrite formation by means of mechanical pressure can be easier.
3. Sodium is less reducing than lithium, meaning that more substances are thermodynamically stable in direct contact with the metal. This can be an important advantage when designing cell concepts including solid ion-conducting membranes. Many Li-ion conducting solid electrolytes degrade exposed to direct contact with metallic lithium [16]. Moreover, by employing beta-alumina, an excellent Na-ion conducting solid electrolyte is commercially available.
4. The total number of known sodium compounds is larger compared to lithium, so cell reactions might require more intermediate steps or stop at a different stoichiometry. Two notable exceptions exist that might be of advantage for sodium cells. Aluminium forms binary alloys with lithium but not with sodium. Therefore, aluminium instead of the more expensive copper can be used as a current collector for the negative electrode in sodium batteries. Another exception that might have practical relevance is that sodium, in contrast to lithium, does not form a stable nitride when exposed to N_2_ atmosphere. This has an immediate impact on Li/O_2_ and Na/O_2_ cells when operated under air.
5. The larger sizes of the sodium atom and ion compared to lithium (+82% for the atom and +25% to +55% for the ion, depending on the coordination) lead to larger volume changes during cycling. Sodium-based electrodes might therefore degrade faster and the formation of stable interfaces might become more difficult. But the smaller size of the lithium ion corresponds to a larger charge density, and the lithium ion polarizes it environment stronger than the sodium ion. This causes severe differences in chemical bonding and ion mobility.
6. The solubility of sodium and lithium compounds in solvents are different. The discharge products and/or interphases (SEI formation) can therefore dissolve to different degrees and electrolyte solutions might have different properties.

### 2 Lithium–oxygen (Li/O_2_) and sodium–oxygen (Na/O_2_) batteries

This section is organized as follows. Firstly, the basic operating principles and energy densities of Li/O_2_ and Na/O_2_ cells are discussed. Secondly, the state-of-the-art knowledge on Li/O_2_ cells is summarized. As several reviews have been published in this field, we will only briefly highlight important achievements and discuss recent developments. Thirdly, the available literature on the Na/O_2_ cell is summarized and similarities and differences to the analogue Li/O_2_ cell are discussed. Li/S_8_ and Na/S_8_ batteries are discussed the same way in chapter 3. The section will end with a brief summary and outlook.

#### 2.1 Operating principles and general remarks

The operating principle of a lithium–oxygen battery is depicted in Figure 2b. The major difference compared to Li-ion batteries is that the battery is designed as an open system that enables uptake and release of atmospheric oxygen at the cathode during cycling (hence the name “lithium–air battery”, which is misleading as mostly pure oxygen gas is used). During discharge, lithium is oxidized at the negative electrode and oxygen is reduced on the positive electrode. Similar to a fuel cell cathode, the positive electrode is a porous, electron-conducting support (gas diffusion layer, GDL) that enables oxygen transport, oxygen reduction (ORR) and oxygen evolution (OER) during cell cycling. Carbon-based materials are mostly used for this purpose. Considering the basic principle of this cell concept, some challenges are immediately obvious: (1) The implementation of special membranes is necessary to prevent contamination of the cell by unwanted gases from the atmosphere (N_2_, CO_2_, and also H_2_O for the case of non-aqueous systems) and to protect the metal electrode from oxygen exposure. At the same time, drying out of the cell due to solvent evaporation must be avoided. (2) The gas transport must be fast enough to enable sufficiently fast discharging and charging. (3) The cell needs to provide enough free volume to accommodate the discharge product.

**Figure 3:**
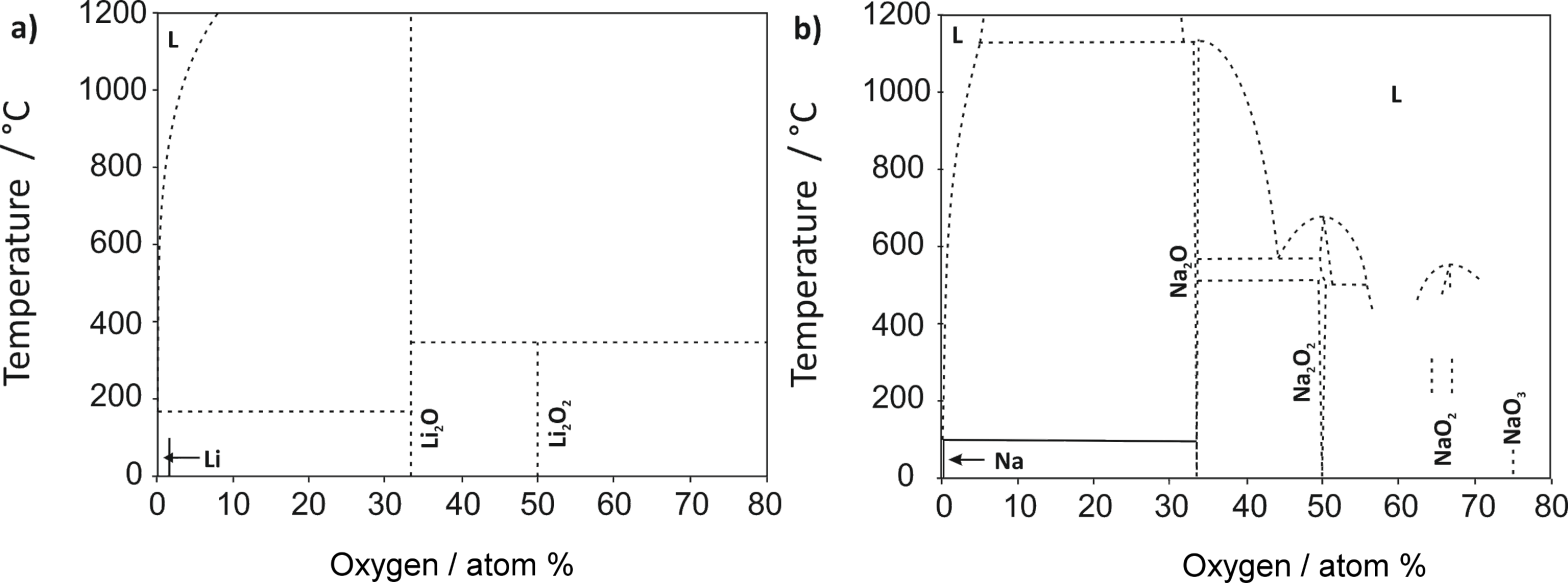
(a) The Li–O phase diagram. (b) The Na–O phase diagram. Figure redrawn based on [18] and [19].

The reaction product depends on the type of electrolyte used. In aqueous electrolytes, water becomes part of the cell reaction and dissolved LiOH is formed during discharge, which precipitates as LiOH·H_2_O once the solubility limit is reached. The need to protect the lithium anode from direct contact with water is experimentally challenging, so most research has been devoted to lithium–oxygen batteries with an aprotic electrolyte. Some possible discharge products can be directly predicted from the Li–O phase diagram shown in Figure 3a. Under ambient conditions, the thermodynamically stable phases are lithium oxide (Li_2_O) and lithium peroxide (Li_2_O_2_). As these compounds are insulators, GDLs with a high surface area are used to improve the kinetics. Two other cell concepts that have been studied to a lesser extent are cells with a mixed aprotic/aqueous electrolyte and cells based on solid electrolytes. A sodium–oxygen battery can be designed exactly the same way but the phase diagram (Figure 3b) shows that in addition to Na_2_O_2_ and Na_2_O, sodium superoxide (NaO_2_) can also be formed (although possibly only kinetically stable under ambient conditions). The relative stability of NaO_2_ was recently calculated by two groups with somewhat controversial results (see the section The sodium–oxygen (Na/O_2_) battery for more details). Sodium ozonide (NaO_3_) has been frequently reported as being unstable under ambient conditions and hence is not considered. Different discharge products may form in alkali-metal–oxygen cells. As will be discussed later in more detail, the discharge products in aprotic electrolytes are Li_2_O_2_ in Li/O_2_ cells, and Na_2_O_2_ and NaO_2_ (and Na_2_O_2_·2H_2_O) in Na/O_2_ cells. It is an open and interesting question whether the relative stability of the different alkali oxides is correctly represented in the phase diagrams, as the influence of water may have been overlooked. It is well known that even small amounts of water can stabilize oxide phases, which are otherwise absent in the phase diagram [17].

The theoretical cell voltages and energy densities of the cell reactions are summarized in Table 1. We note that also potassium-oxygen batteries are being studied [20–21]. The energy densities however, are lower. The values for energy densities vary depending on whether the weight of oxygen is included or not, but all metal–oxygen batteries are superior compared to Li-ion batteries in terms of theoretical energy capacity. This is also the case for cells with NaO_2_ as a discharge product, although they are based on one-electron transfer. It is important to note that all values in Table 1 are theoretical values. As the concept of metal–oxygen batteries requires many additional design-related components (e.g., gas diffusion layer, membranes to minimize oxygen diffusion towards the metal anode and to minimize access of other detrimental gases from the atmosphere) the weight penalty for reaching a commercial product will be much higher as compared to LIBs. The estimated values of the practical energy density vary greatly. Values of 1700 Wh/kg at the cell level and 850 Wh/kg at the battery level have been suggested by Girishkumar et al. [22], while Christensen et al. estimated around 1300 Wh/kg for the cell level [23]. PolyPlus, one of the leading companies working on lithium–air batteries, project 600 Wh/kg and 1000 Wh/L, respectively [24]. Recently, Gallagher et al. comprehensively studied the use of Li–air batteries for electric vehicles (EVs) and predicted values of around 250–500 Wh/kg and 300–450 Wh/L on the system level. The authors concluded that Li–air batteries will not be a viable option for commercial automotive applications [25], which then also would exclude Na–air systems. An additional challenge for electric vehicle application is that the current densities of lithium–oxygen cells (usually below 1 mA/cm^2^) are still too small and an improvement by one to two orders of magnitude is necessary, as the target current density should be in the range of 8–80 mA/cm^2^ [23,26]. Although these estimates depend on the assumptions made, it is clear that the competition between lithium–oxygen batteries and LIB technology will depend on the application. In any case, the limits of such a technology will only be fully apparent once a meaningful prototype has been built. The only report of a fully engineered cell reported in the literature is given by PolyPlus for a primary, aqueous, lithium–air battery. Their cells with a total capacity of about 10 Ah achieved 800 Wh/kg at a current density of 0.3 mA/cm^2^ [24]. Given the fact that research on rechargeable lithium–oxygen cells is still at a more fundamental level, possible applications should therefore not be restricted to EVs.

**Table 1:** Theoretical cell voltage, *E°*, gravimetric and volumetric energy density, *W*_th_, and charge density *Q*_th_ for lithium–oxygen and sodium–oxygen batteries with a metal anode. Values for the gravimetric energy densities are given without and including the weight of oxygen. All other values given refer to the discharged state. Thermodynamic data derived from HSC chemistry for all compounds in their standard state at 25 °C. Calculations for the aqueous systems are simplified and assume formation of hydrated hydroxide with all water resulting from the electrolyte.

Cell reaction	*E°* / V	*W*_th_ / Wh/kg	*Q*_th_ / mAh/g	*W*_th_ / Wh/L	*Q*_th_ / mAh/cm^3^

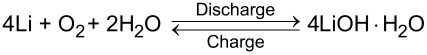	3.40	2684 / 2172	639	3280	1634
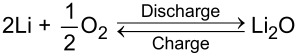	2.91	11229 / 5216	1794	10501	3606
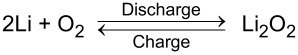	2.96	11421 / 3456	1168	7983	2698
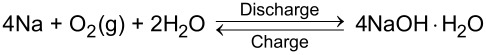	2.77	1486 / 1281	462	–	–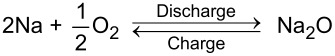	1.95	2273 / 1687	867	3828	1968
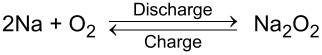	2.33	2717 / 1602	689	4493	1936
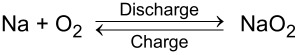	2.27	2643 / 1105	488	2431	1074
Li-ion (average cathode vs Li/Li^+^)	3.8	530	140	2300	600

For sodium cells, the theoretical energy densities are smaller compared to the analogue lithium systems. Therefore, the development of a high energy device might be more challenging unless the sodium cell chemistry provides specific advantages which might include: (1) faster kinetics of the oxygen electrode in the case of NaO_2_ as a discharge product, (2) a higher tolerance against atmospheric nitrogen as no stable nitride exists, (3) cell concepts with a molten sodium electrode [26], or (4) the availability of beta-alumina as a solid electrolyte that might enable cell concepts including solid membranes.

Considering all of these aspects, lithium–oxygen and sodium–oxygen batteries are very attractive means for energy storage in theory, but the development of practical cells is an ambitious goal. Even in the best scenario, such materials are unlikely to be developed for EV applications. However, the major showstopper for the development of rechargeable alkali–air devices is that the cell systems usually suffer from severe side reactions that hinder stable cell cycling for a large number of cycles. As will be discussed below, the sodium–oxygen cell indeed shows some promising advantages over the lithium system but several fundamental challenges must be understood and solved before the development of a practical battery might become feasible.

#### 2.2 Classification of voltage profiles

**Figure 4:**
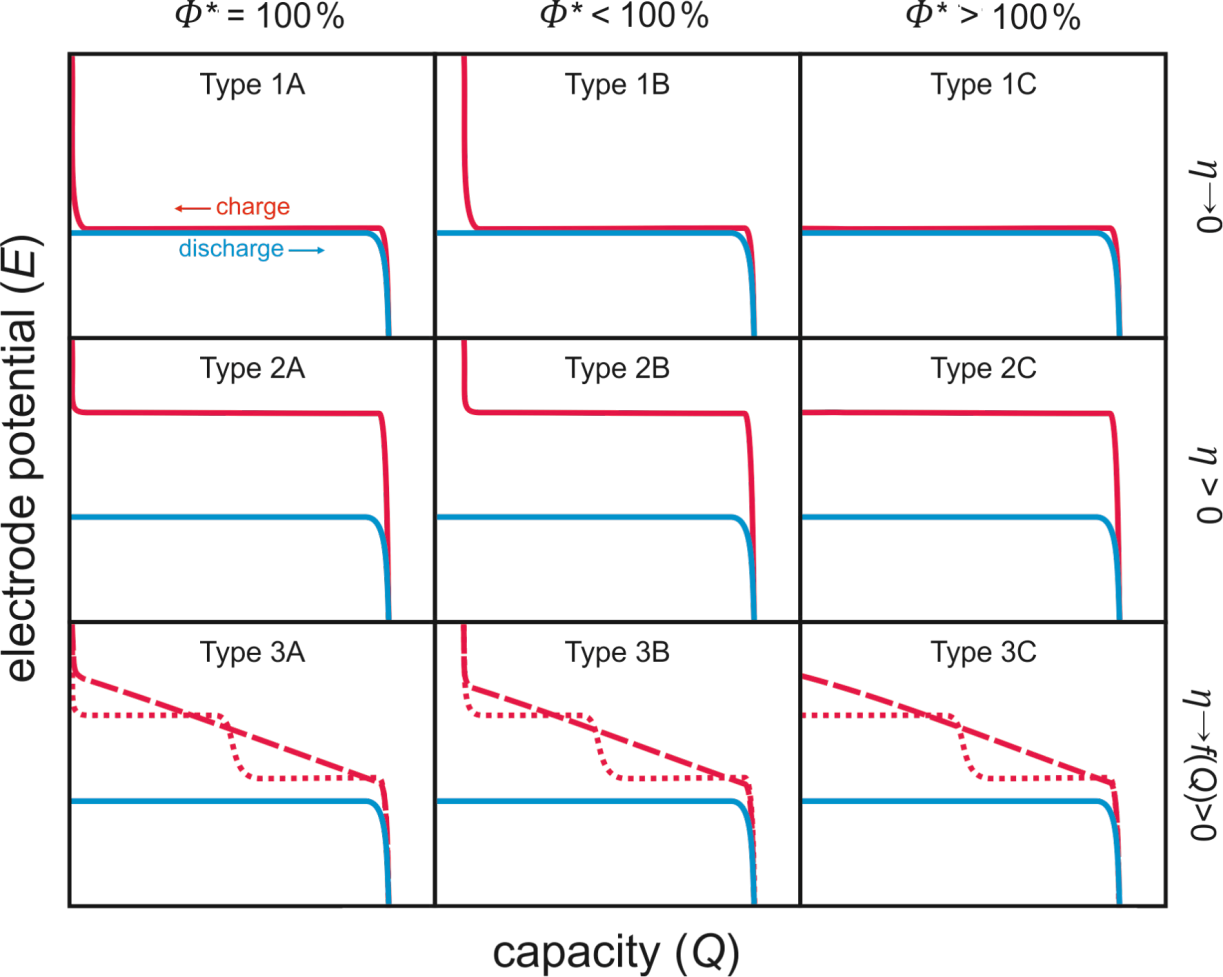
Matrix for classifying voltage profiles of metal–oxygen batteries. Type 1A is the ideal case. Frequently observed are Type 1B, 2C, 3B and 3C. The coulombic efficiency is calculated as Φ = *Q*_charge_/*Q*_discharge_ × 100%.

The basic properties of a cell reaction can be easily discerned from diagrams showing the voltage profiles (discharge/charge curves) as their shape provides direct information on the complexity, reversibility and efficiency of the cell reactions. At moderate currents, most of the Li/O_2_ and Na/O_2_ batteries show quite similar discharge curves: the discharge voltage is more or less constant and comparably close to the theoretical cell potential. The discharging stage ends with a sudden potential drop (“sudden death”). The charging curves, however, vary significantly and heavily depend on the cell configuration (sodium or lithium cell, type of electrolyte, use of catalysts, type of GDL, etc.). So in order to more easily discuss the experimental results, the classification of the voltage profiles according to the shape of the charging curves is useful (Figure 4).

The starting point of the matrix is the ideal cell reaction, classified as Type 1A. The voltage profile is characterized by negligible overpotentials for discharge and charge and a Coulombic efficiency of Φ = 100%, that is, the charging voltage is close to its theoretical value and charging ends with a sudden increase in cell potential as soon as all discharge products are decomposed. Based on this ideal cell reaction, the following matrix can be derived.

**Type 1:** The combined overpotentials (sum of the overpotentials during discharge and charge) approach zero, meaning that kinetic limitations are negligible.

A: Coulombic efficiency = 100%. The cell reaction is completely reversible. B: Coulombic efficiency < 100 %. The reaction is only partially reversible. Possible reasons are that some of the discharge product became electrochemically inactive, lost contact to the electrode, or underwent irreversible side reactions with other cell components. C: Coulombic efficiency > 100 %. Either electrochemical side reactions or a so-called shuttle process (chemical shortcut) between both electrodes takes place. A shuttle process can be intentional (e.g., overcharge protection in LIBs) or unintentional (e.g., polysulfide shuttle in lithium–sulfur batteries). Unless it is intentional, Coulombic efficiencies exceeding 100% are always a sign of undesired side reactions. Note that in this case the Coulombic efficiency of the desired cell reaction is also below 100%. Values exceeding 100% simply arise from the fact the shuttling/side reactions give rise to additional external currents leading to charging capacities exceeding the discharge capacities.

**Type 2**: Considerably high combined overpotential occurs and the cell kinetics are sluggish. Various processes can contribute to overpotential, but using catalysts or optimizing the transport properties might be effective strategies for improvement.

**Type 3:** The voltage continuously increases during charging and might exhibit additional plateaus. Such a behavior indicates a more complex electrode reaction. In most cases, this is a strong indication of undesired side reactions. Additional plateaus during charging can originate from the electrochemical decomposition of side products stemming from undesired side reactions between cell components and the discharge product. For example, Li_2_O_2_ can react with the electrolyte to form Li_2_CO_3_, which decomposes during charging at high voltages. Another possibility is that the cell discharge was incomplete (e.g., the discharged state is a mixture of Na_2_O_2_ and NaO_2_) and the different discharge products decompose at different potentials during charging.

The matrix certainly includes some simplifications: side reactions might be time dependent, the voltage profile can change during cycling, overpotential increases with current density, etc. However, the matrix allows for a straightforward classification of the large number of different experimental results published. Briefly, the more different the voltage profile is from the ideal case (Type 1A), the more challenges that have to be tackled to achieve a reversible cell reaction. So far most metal–oxygen batteries show the following behavior when cycled at moderate rates: Type 1B is found for Na/O_2_ cells with NaO_2_ as discharge product. Type 2C, 3B, and 3C are found for Li/O_2_ and Na/O_2_ cells with either Li_2_O_2_, Na_2_O_2_, or Na_2_O_2_·2H_2_O as a discharge product.

It is important to note that values for the capacity, *Q*, of metal–oxygen cells are presented differently as it is usually done. The common way in battery research is to state the capacity in mAh per gram of active material, that is, per gram of LCO or sulfur, for example. This is possible because the electrode contains all active material and the battery is a closed system. In open metal–oxygen batteries, the active material (oxygen) is not part of the electrode and the discharge product forms as a new phase during discharge. Therefore, capacity values are usually given in mAh per gram of carbon support. As the absolute amount of carbon used is usually very small, the reported capacity values can reach very high numbers, easily exceeding 1000 mAh/g. Stating this value only, however, is clearly not sufficient to judge the performance of the cell and may easily mislead the uninformed reader [22,27]. At a minimum, carbon loading (mg/cm^2^), electrode size and thickness of the carbon layer (if known) and the total amount of charge should be stated. Given this, the charge density (mAh/cm^3^) and areal capacity (mAh/cm^2^) can be calculated and benchmarked against commercialized LIB materials (approximately 1–4 mAh/cm^2^ and 350–600 mAh/cm^3^). A comparable problem is that the common definition of the C rate cannot be applied to metal–oxygen cells without further assumptions, and therefore, discharge and charge rates are usually given as current density (calculated by using the cell cross section).

#### 2.3 State-of-the art and recent developments

**2.3.1 The lithium–oxygen (Li/O_2_) battery:** In 1969, A. E. Lyall filed a patent application on “A room-temperature-operated fuel cell comprising an oxygen electrode, a lithium metal-containing electrode, and an electrolyte comprising an inert, aprotic organic solvent […], which contains an inorganic or organic ionizable salt […]” [28]. Interestingly, the components of this Li/O_2_ battery are remarkably close to those utilized today. The pioneering work on rechargeable, room temperature, Li/O_2_ batteries with a non-aqueous electrolyte can be summarized as follows. In 1996, Abraham et al. reported on “A polymer electrolyte-based rechargeable lithium/oxygen battery” [29]. This cell could be re-charged at room temperature at least three times at potentials as low as 3.8 V. In 2002, Read characterized a Li/O_2_ cell comprising different carbon materials and different electrolyte formulations [30]. This was the first work to analyze and correlate the amount of consumed gaseous oxygen with respect to the transferred electric charge, and found that this value varies strongly depending on electrolyte composition. As will be discussed in the following sections, this kind of characterization is crucial for both evaluating and understanding aprotic Li/O_2_ cells. He interpreted this variation using mixtures of Li_2_O_2_ and Li_2_O which are formed during discharge.

Today’s strong interest in Li/O_2_ batteries was most likely initiated by the work of Bruce et al. who reported on a Li/O_2_ cell in 2008 that could be efficiently cycled, resulting in capacities as high as 3000 mAh/g_carbon_ by introducing α-MnO_2_ nanowires as catalyst in the oxygen cathode [31]. From 2008 onwards, the number of publications on Li/O_2_ batteries rapidly increased. The progress in Li/O_2_ research and development is the subject of numerous review articles [22,32–34]; therefore, we focus here on a brief summary of, in our opinion, the major trends in current research efforts.

**Figure 5:**
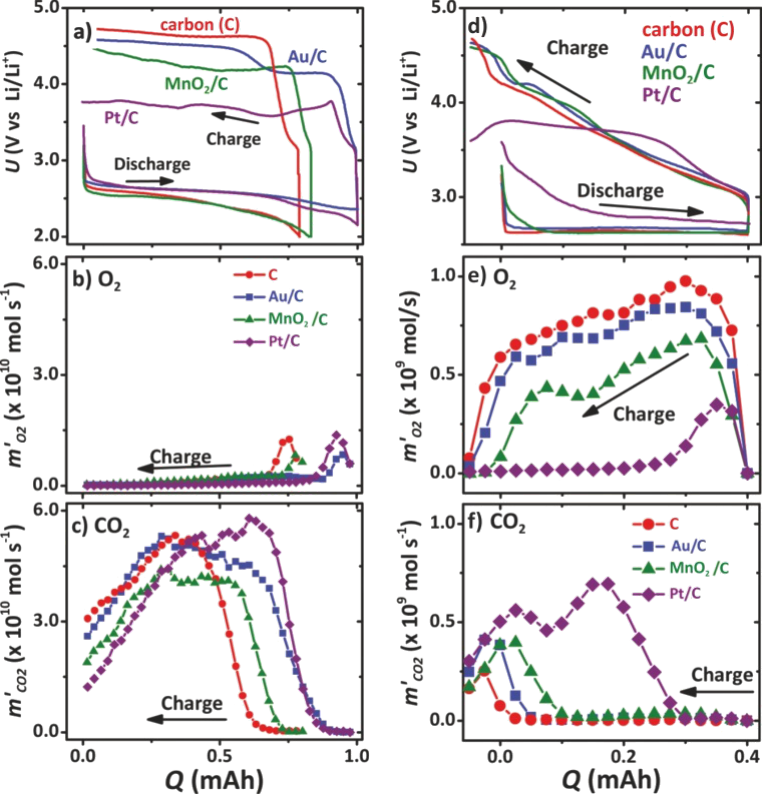
DEMS analysis of Li/O_2_ cells with different electrolyte compositions, namely a mixture of propylene carbonate and dimethoxyethane, PC:DME, (a–c) and pure dimethoxyethane, DME, (d–f). Furthermore, gold, platinum and manganese dioxide were tested as heterogeneous catalysts. (a) and (d) show the galvanostatic cycling characteristics. (b) and (d) show the desired oxygen (O_2_) evolution during charging, and (c) and (f) show the corresponding carbon dioxide (CO_2_) evolution measured. Figure adapted with permission from [42], copyright 2011 American Chemical Society.

2.3.1.1 Catalysts: As shown by Bruce et al., Li/O_2_ cells with liquid aprotic electrolyte can apparently be recharged, but rather high potentials (>4 V vs Li/Li^+^) for the decomposition of Li_2_O_2_ (OER) are required. Hence, research focused on the preparation and characterization of catalytically active materials for Li/O_2_ cells is aimed at higher discharge capacities and lower overpotentials during cycling. Various metal oxide materials, mostly manganese oxides (MnO_2_, Mn_3_O_4_), but also others have been proposed [9,31,35–38] as well as noble metals [39–41]. In 2011, McCloskey et al. attentively figured out that catalysts such as Pt, MnO_2_ or Au also promote the decomposition of the aprotic electrolyte rather than the oxygen evolution reaction (see also Figure 5) [42]. Although both the functionality and the necessity of heterogeneous catalysts in Li/O_2_ cells remain unsolved, the search for improved heterogeneous catalysts for improved cyclability is still the subject of many new articles on Li/O_2_ batteries. The most promising catalyst material, ruthenium nanocrystals, was reported by Sun et al., and the cells show a type 3A hysteresis (see Figure 4) with a charge potential as low as 3.5 V [41].

2.3.1.2 Electrolyte instability: Liquid aprotic electrolytes containing carbonate-based solvents such as propylene carbonate (PC), ethylene carbonate (EC), diethyl carbonate (DEC), or dimethyl carbonate (DMC) have been applied in almost all of the experimental studies on catalyst materials between 2006 and 2010, because these compounds are well used in LIBs. A comprehensive overview of the properties of liquid lithium electrolytes is given in [43]. In the beginning, only minor attention had been paid to clarify the chemistry taking place in the cells, for example by analyzing all chemical species being formed during cycling. In 2010 Mizuno et al. reported an FTIR and TEM study of the reaction products in Li/O_2_ cells employing a PC based electrolyte [44]. They concluded that, although the cell was cycled up to 100 times, lithium carbonate (Li_2_CO_3_) and lithium alkyl carbonate species (RO–(C=O)–OLi) instead of Li_2_O_2_ were found as discharge products. In the following, similar observations for various carbonate-based solvents were reported by other groups as well [45–48]. The experimental findings are supported by computational studies looking into molecule stability and possible decomposition pathways for the solvents [49–50] and it is now clear that carbonate-based electrolytes are not suitable for aprotic Li/O_2_ cells. In addition it was found that many electrolyte salts are at least partially decomposed during cell cycling as well [47,51–53]. From this perspective, it is of note that even as early as 1991, Aurbach et al. reported the irreversible decomposition of propylene carbonate (PC) in the presence of oxygen during cyclic voltammetry experiments [54].

2.3.1.3 Stable electrolytes: The finding that the decomposition of the carbonate solvents was responsible for much of the capacity in Li/O_2_ cells was a setback that quickly changed the research focus to the stability and potential decomposition reactions of the electrolyte components. Three different reactive oxygen species may be involved in solvent decomposition reactions: (a) molecular oxygen (O_2_), (b) superoxide (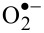, “LiO_2_”) and (c) peroxide species (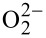, Li_2_O_2_). The individual role of these different species in the decomposition reactions is still unclear. In a number of studies on different solvents have been made including ionic liquids [55–57], sulfoxides (DMSO) [58–60], amides [61–62], and others [62–64]. The ether-based glyme solvents with the general structure CH_3_–O–(CH_2_–CH_2_–O)*_n_*–CH_3_ with *n* = 1–4 are the current state-of-the-art solvents [65–69], although they are not entirely stable. A solvent with better performance still must be found. Adams et al. recently reported on a chemically modified monoglyme (DME), 2,3-dimethyl-2,3-dimethyoxybutane, as a promising solvent as it leads to a significantly lower CO_2_ evolution (see DEMS) and lower overpotentials for both discharge and charge [70]. Analogous to the lithium–sulfur batteries, the use of lithium nitrate (LiNO_3_) seems to improve the cyclability of Li/O_2_ cells as well. In publications by Liox Power Inc., it was shown that LiNO_3_ leads to an improved stability of the lithium electrode solid electrolyte interphase (SEI) formation [61]. Kang et al. showed that it also leads to an improved stability of carbon at the cathode [71].

2.3.1.4 Differential electrochemical mass spectrometry (DEMS) studies: The electrolyte decomposition is a major drawback that made DEMS studies inevitable in Li/O_2_ cell research. Today, this real-time analysis of the gaseous species being consumed or released during cell cycling is a necessary standard technique. In an ideally operating cell, only oxygen (O_2_) evolves during recharge, but in reality, other products such as CO_2_, H_2_O or H_2_ are detected and give evidence for unwanted side reactions. Therefore, DEMS or online electrochemical mass spectrometry (OEMS) was introduced into the Li/O_2_ battery field and is now one of the most important, but seldom employed, diagnostic tools of current research [46,72–77]. Figure 5 shows the potential of DEMS analysis when comparing different electrolyte and oxygen electrode materials in an Li/O_2_ cell [42]. Figure 5a,d shows the galvanostatic cycling characteristics for a PC:DME electrolyte and a pure DME electrolyte, respectively. For both electrolytes, in addition to a pure carbon electrode, heterogeneous catalysts, such as Pt, Au and MnO_2_ were also tested. It was shown that the catalysts (especially in combination with the PC:DME electrolyte) lead to a significant reduction of the charge overpotential, and in the case of Pt, by almost 1 V in comparison to pure carbon. However, the corresponding DEMS data in Figure 5b,c clearly prove that only minor amounts of oxygen (O_2_) but mainly CO_2_ is evolved during the charging of the cell. Thus, by means of DEMS, McCloskey et al. could clearly prove that the improved rechargeability due to the heterogeneous catalysts is not related to an improvement of the Li_2_O_2_ decomposition, but rather to the promotion of the electrolyte decomposition. In contrast, in pure DME electrolyte, oxygen evolution is indeed observed. However, in this case, the catalyst materials had almost no impact on the charge overpotential, but again only led to an increased evolution of CO_2_.

2.3.1.5 Number of electrons per oxygen molecule, e^−^/O_2_: As already mentioned above, Read observed that in certain electrolytes the oxygen consumption during discharge was too low for the sole formation of Li_2_O_2_ and proposed that Li_2_O is formed in concomitance [30]. Looking back to these results, one can now definitively assume that Read observed the partial decomposition of the electrolyte during discharge rather than the formation of Li_2_O species. Hence, it is of crucial importance to understand that for metal–oxygen cells the reversibility cannot be proven by solely stating Coulombic efficiencies. It is, as introduced by Read, the ratio between consumed or released oxygen and the amount of transferred charge that gives the true reversibility. For an ideal Li/O_2_ cell, where Li_2_O_2_ is reversibly formed, two electrons are transferred for each reacting oxygen molecule, or 2.16 mAh for 1 mL of gaseous oxygen at 298 K and 10^5^ Pa. Any deviation from this ratio is a strong indication for (partial) malfunction and hence, this value is essential, especially when new electrolyte or electrode components are tested. A simple but effective way to measure this ratio is the usage of a pressure sensor and a hermetic gas reservoir as introduced by McCloskey et al. [46,78] or via quantitative DEMS/OEMS, which in addition allows for the identification and separation of the gaseous reactants [42,60,66,68,74]. In addition to the analysis of gaseous reactants, first attempts are also made to quantify the amount of discharge product formed [67,78–80]. This will also be an important step towards true reversibility evaluation.

**Figure 6:**
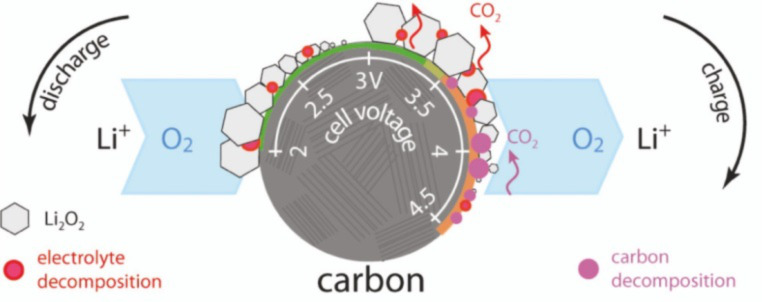
Sketch by Thotiyl et al. illustrating their findings on the oxidation of the carbon electrode. At discharge at potentials below 3 V, mostly Li_2_O_2_ is electrochemically formed and accompanied by electrolyte decomposition. During recharge at potentials between 3.0 and 3.5 V, CO_2_ evolution is mainly related to electrolyte decomposition. Lastly, at potentials higher than 3.5 V, oxidation of the carbon electrode takes place. Figure adapted with permission from [83], copyright 2012 American Chemical Society.

2.3.1.6 Electrode materials: Obviously a Li/O_2_ cell is a very reactive environment and it seems likely that the different oxygen species would also react with other components of the oxygen electrode. Black et al. exposed battery components to potassium superoxide dissolved in aprotic liquids and found that polyvinylidene fluoride (PVDF), a common binder material, decomposes while lithium fluoride (LiF) is formed [81]. They suggest that LiO_2_, a strong base that is formed as an intermediate in a Li/O_2_ cell, extracts protons from the PVDF polymer. From the thermodynamic point of view, carbon is also reactive towards, for example, Li_2_O_2_ or oxygen at high oxidative potentials, too. For this purpose McCloskey et al. employed a ^13^C carbon electrode and monitored CO_2_ species via DEMS evolved during the charge process [82]. The appearance of ^13^CO_2_ at the end of the charge process was taken as evidence for carbon oxidation. Similar findings were made by Thotiyl et al. (Figure 6) who proposed that carbon oxidation can be avoided as long as potentials remain below 3.5 V vs Li/Li^+^ [83]. The same group also investigated non-carbon electrodes, such as nanoporous gold or titanium carbide (TiC) [60,84]. Both materials are claimed to significantly improve the cycle performance compared to carbon electrodes due to a higher chemical stability towards lithium oxide species. On the other hand, the solvent employed in their study (DMSO) is known to be unstable in Li/O_2_ cells [85–86]. Notwithstanding the above, the understanding of electrode corrosion and the search for stable electrode materials, either modified carbons or non-carbon materials, is of crucial importance for a reliable Li/O_2_ battery.

**Figure 7:**
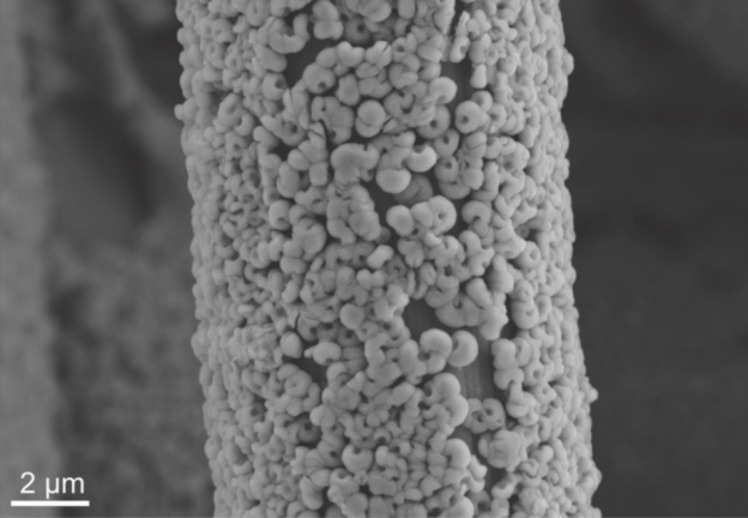
SEM image of toroidal Li_2_O_2_ nanoparticles on a carbon fiber (10 µm in diameter) that form as a discharge product in lithium–oxygen cells (C. L. Bender, JLU Giessen).

2.3.1.7 Particle growth and dissolution: At first glance, the chemistry of a Li/O_2_ cell may appear quite simple, however, due to worldwide research efforts within the last four years, it was recognized that it is in fact, a very complex cell chemistry. As a consequence it was necessary to refocus on fundamental aspects such as the growth and dissolution process of Li_2_O_2_ particles during cycling on a microscopic scale. Various morphologies of Li_2_O_2_ deposits are reported in literature. On the one hand, so-called Li_2_O_2_ “donuts” or toroids are reported that form to a diameter of up to 1 µm, depending on solvent and cycling conditions (see Figure 7). On the other hand, thin film coverage of the carbon electrode is found. It is reported that at low current densities large toroid-like particles form and that at high current densities Li_2_O_2_ film formation takes place [32,87]. Interestingly, Read basically made the same observation in 2002 and concluded that large particles could only grow if the oxide (Li_2_O_2_) is (a) soluble in the electrolyte (b) able to migrate on electrode surface or (c) capable of catalyzing the oxygen reduction [30]. Theoretical studies are particularly focused on possibility (c) and look for electric transport in Li_2_O_2_. Since Li_2_O_2_ is an intrinsic wide band gap insulator, additional transport mechanisms such as transport along metal-type surfaces or hole polaron transport are proposed [88–91]. The assumption of a soluble redox-active species (e.g., soluble O_2_^−^), as polysulfides in the case of lithium–sulfur or sodium–sulfur batteries, has only very recently been seriously taken into account. Viswanathan et al. suggest that Li_2_O_2_ grows only to film deposits of 5–10 nm in thickness because charge transport through the Li_2_O_2_ layer can only proceed by hole tunneling [92–93]. In a very recent study they propose that the comparably large donut structures can only be observed in the presence of water in the electrolyte, which leads to soluble superoxide species [94]. Their findings, however, are in contrast to those of Zheng et al. who were able to operate a model all-solid-state Li/O_2_ cell, without any liquid electrolyte, in an environmental SEM and observed the formation of large toroid particles larger than 500 nm [95]. To conclude, even the dissolution process of Li_2_O_2_ during battery operation is not fully understood and continues to be a part of research efforts.

**Figure 8:**
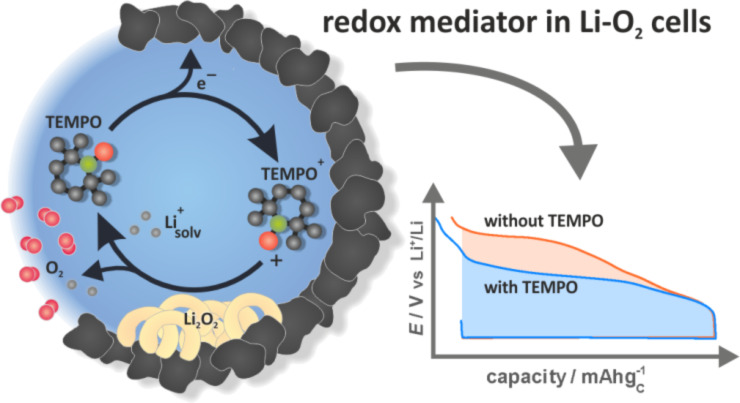
Illustration of TEMPO as a redox mediator (RM) in an Li/O_2_ cell reversibly catalyzing the Li_2_O_2_ oxidation. Figure adapted with permission from [99], copyright 2014 American Chemical Society.

2.3.1.8 Electrolyte additives: The electrochemical activity of Li_2_O_2_ itself is quite poor without doubt, especially for the charge process (OER). Hence catalysis is necessary especially when aiming for experimental current densities. The results of heterogeneous catalysts until now did not fulfill the expectations. A new and promising concept is to add soluble and redox-active molecules to the liquid electrolyte. In 2011 Liox Power Inc. filed a patent application on such “soluble oxygen evolving catalysts for rechargeable metal–air batteries” [96]. Those often called redox mediators (RM) molecules possess a redox potential higher than that of Li_2_O_2_ (*E*°_RM_ > *E*°_Li2O2_ = 2.96 V vs Li/Li^+^). During recharge of the battery the RM molecules are oxidized at the oxygen electrode. Subsequently, the oxidized RM molecules oxidize Li_2_O_2_ chemically and hence catalyze the OER. In 2013 Chen et al. reported on tetrathiafulvalene (TTF) as RM with redox potentials, TTF/TTF^+^ and TTF^+^/TTF^2+^, of 3.4 to 3.7 V. With TTF in a DMSO:LiClO_4_ electrolyte the Li/O_2_ cells showed a Type 1C hysteresis and significantly improved kinetics for the charge process. In addition e^−^/O_2_ ratios very close to two, as expected for Li_2_O_2_ oxidation, were claimed [97]. Also lithium iodide [98] and TEMPO [99] have been recently studied as RMs with promising results (see Figure 8). It is worth noting that redox mediators (also called “relays”) are used also in other applications for the improvement of poor electrode kinetics.

An interesting and complementary approach is to increase the solubility of oxides species (e.g., Li_2_O_2_) in the liquid electrolyte which would allow fast transport of oxide species to active electrode sites. Lim et al. synthesized TFSI based cations that are able to considerably increase the solubility of Li_2_O_2_ in DMSO [98], and Lopez et al. reported on hexacarboxamide cryptands that are capable of incorporation of peroxide dianions in solution [100]. As these approaches are quite new, several questions such as long term functionality and stability of the molecular additives in an Li/O_2_ battery need to be investigated. Nevertheless, we believe that major improvements are possible due to chemical tailoring of the molecules with respect to desired functionality.

In conclusion, several challenges for the development of aprotic Li/O_2_ cells with competitive performance remain. Within the last few years more and more researchers focus on the chemical processes taking place during operation of metal–oxygen batteries, which surely will lead to deeper understanding of Li/O_2_ batteries and its potential in application. This is remarkable, especially in the fast moving field of battery research, as experimental mechanistic studies are usually time demanding and require both a careful execution of experiments and the use of complex and often expensive analytical methods.

**2.3.2 The sodium–oxygen (Na/O_2_) battery:** The sodium–oxygen battery is based on the same cell concept as the lithium–oxygen battery, however, only very little literature is available. Mostly aprotic electrolytes have been used and only one study on a mixed aprotic/aqueous electrolyte has been published. This may be due to the strong reactivity of sodium with water. Although research on Na/O_2_ cells started only in 2010 the number of publications now rapidly increases. To date, more than 20 studies have been published altogether. The currently most striking characteristic of aprotic Na/O_2_ cells is that, in contrast to Li/O_2_ cells, a number of different discharge products have been reported: sodium superoxide (NaO_2_), sodium peroxide (Na_2_O_2_), sodium carbonate (Na_2_CO_3_), hydrated sodium peroxide (Na_2_O_2_∙2H_2_O) and sodium hydroxide (NaOH). The underlying reason for this is not clear yet but it might be also related to the different experimental conditions used in the different studies. A summary of selected experimental parameters and reported discharge products is shown in Table 2.

**Table 2:** Literature overview on Na/O_2_ cells summarizing experimental conditions and reported discharge products.

Reference	Cathode composition	Electrolyte	Discharge product	Verified by	Max. dis. capacity / mAh/g	Type^a^

Peled et al. [26]	E-TEK air electrode, 10% Pt support, XC72 coated with Na_2_CO_3_	0.1 M calixpyrrole, 1 M NaClO_4_ in PEGDME/PC (90:10) + 1 wt % Al_2_O_3_	Na_2_O_2_ (assumed)	–	–	2C
Sun et al. [101]	Diamond-like carbon thin film	1 M NaPF_6_ in EC:DMC 1:1	Na_2_O_2_ (Na_2_CO_3_)	FTIR , SAED	3600	2C
Das et al. [102]	Super P	1 M NaClO_4_ in tetraglyme 0.75 M NaOTf in EMIM OTf	Na_2_O_2_ (O_2_) / Na_2_CO_3_, Na_2_C_2_O_4_ (O_2_ + CO_2_)	FTIR , XRD	1390 (O_2_) / 183 (CO_2_) / 3500 (40% CO_2_)	–
Liu et al. [103]	Graphene nanosheets	0.25 M NaPF_6_ in DME 0.25 M NaClO_4_ in DME	Na_2_O_2_	SAED	9268	2C
Li et al. [104]	Graphene nanosheets and nitrogen-doped graphene nanosheets	0.5 M NaOTf in diglyme	Na_2_O_2_	XRD	8600	3B
Liu et al. [105]	NiCo_2_O_4_ nanosheets on Ni foam	1 M NaClO_4_ in DME	Na_2_O_2_	FTIR, SAED	1762	3B
Kim et al. [106]	Ketjenblack	1 M NaClO_4_ in PC, 1 M NaClO_4_ in tetraglyme	Na_2_CO_3_ Na_2_O_2_ · 2H_2_O / NaOH	FTIR, Raman, XRD	2800 (PC) / 6000 (4G)	2C
Jian et al. [107]	CNT paper	0.5 M NaOTf in diglyme, 0.5 M NaTFSI in tetraglyme	Na_2_O_2_ · 2H_2_O	Raman, XRD	7530	3B
Yadegari et al. [108]	Carbon black N330 / NH_3_ or CO_2_ treated	0.5 M NaOTf in diglyme	Na_2_O_2_ · 2H_2_O / little NaO_2_	FTIR, XRD	2873	3C
Hartmann et al. [109]	Gas diffusion layer H2315	0.5 M NaOTf in diglyme	NaO_2_	Raman, XRD	300	1B
Hartmann et al. [78]	GDL H2315	0.5 M NaOTf in diglyme	NaO_2_	Raman, XRD	490	1B
Hartmann et al. [110]	GDL H2315	0.5 M NaOTf in diglyme	NaO_2_	Pressure monitoring	280	1B
McCloskey et al. [67]	P50 Avcarb carbon paper	0.2 M NaOTf in DME	NaO_2_	–	–	1B
Bender et al. [27]	GDL H2315, Ketjenblack, etc.	0.5 M NaOTf in diglyme	NaO_2_	XRD	4000	1B
Zhao et al. [111]	Vertically aligned carbon nanotubes (VACNTs)	0.5 M NaOTf in tetraglyme	NaO_2_	SAED, XRD	4200	1B/3B

^a^See Figure 4 for graphical representations of the different types.

**Figure 9:**
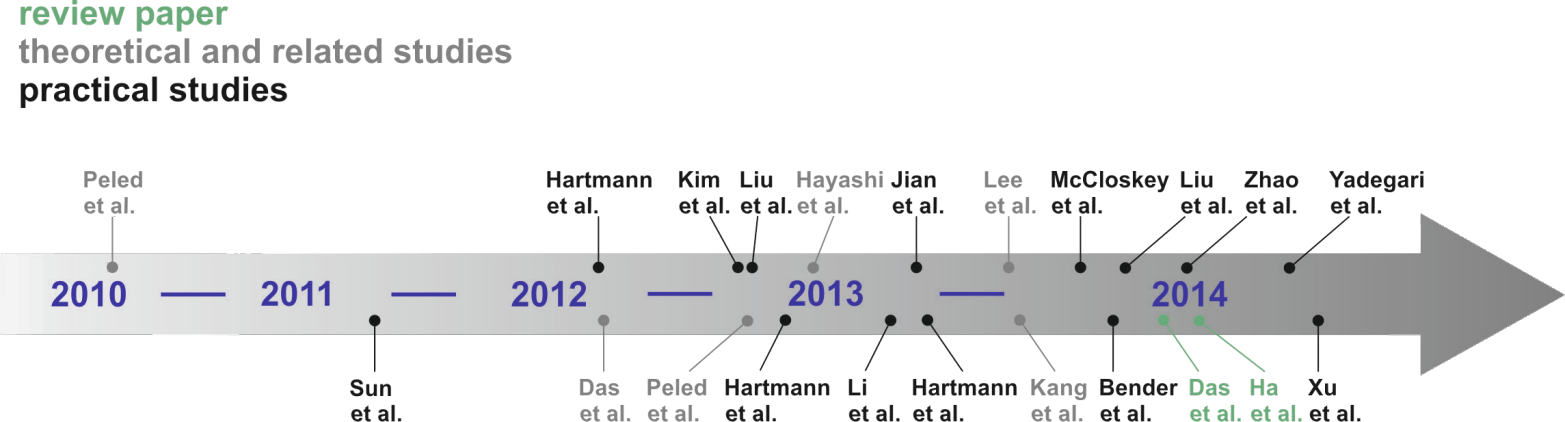
Literature timeline of research papers on aprotic sodium–oxygen batteries (ranked after date of acceptance).

Figure 9 shows a literature timeline of all studies on sodium–oxygen cells. Most of them report on the general cell chemistry and performance improvements in terms of capacity and cycle life. Some related studies including carbon dioxide assisted cells or high temperature cells are also included. These reports are shown in grey and will be discussed at the end of this literature survey. Also two review papers by Das et al. [112] and Ha et al. [113] have been very recently published.

Peled et al. were the first to publish an electrochemical cell based on the reaction of sodium with oxygen in 2010 [26]. The cell was adopted from a fuel cell design and consisted of a molten sodium electrode, a polyglyme/PC (90:10) based electrolyte with different additives and a Pt containing carbon electrode. The cell operated at 105–110 °C. The high temperature concept with molten anode was chosen for several reasons: Counteracting the sluggish cathode reactions, lowering the cell impedance, eliminating dendrites and minimizing interference with water and carbon dioxide. On the other hand, the high reactivity towards the electrolyte was an issue. The cell discharged at 1.75 V (100 µA) and was charged at 3.0 V (50 µA). The discharge product of a full discharge was assumed to be sodium peroxide without further proof by analytical techniques. Later on, the same group published a follow-up study with the main focus on investigating SEI formation and sodium plating/stripping in an ionic liquid based electrolyte [114]. Na_2_SO_4_ was added to the electrolyte as SEI former. Although sodium plating/stripping was obtained for 300 cycles without internal shortcuts, the efficiency with around 70–80% was still unsatisfying. In general, these results underline that studying the reversibility of the ORR/OER reactions in metal–air batteries is not sufficient as also plating/stripping of the alkali metal needs to be reversible in order to achieve a long cycle life. Cell discharge using this IL based electrolyte at 25 µA/cm^2^ was characterized by a sloping decrease, charging (250 µA/cm^2^) mainly occurred at about 3 V. As we will see in the following, the overall cycling behavior of this cell is very different from cells operating with a solid sodium anode at room temperature.

**Figure 10:**
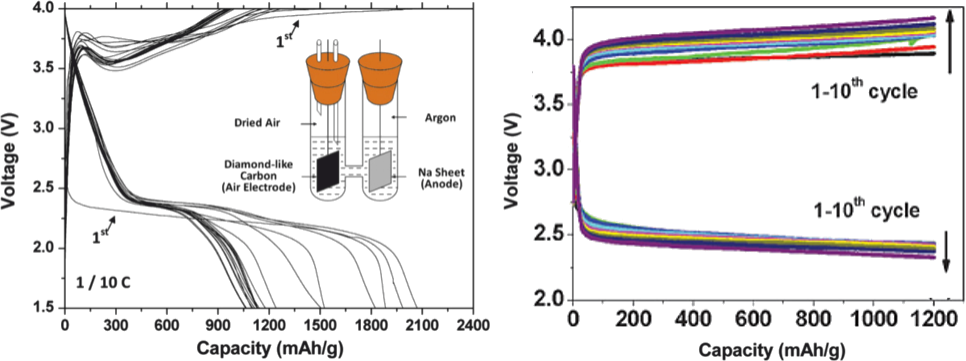
Sketch of the first room temperature sodium–oxygen cell and its discharge and charge potentials during the first ten cycles (left), Figure adapted with permission from [101], copyright 2012 Elsevier B.V. The voltage profile can be classified as Type 2C. Voltage profile of a sodium–oxygen cell with graphene nanosheets as cathode and NaPF_6_ in DME as electrolyte (Type 2C) (right). The discharge product was identified as sodium peroxide. Figure adapted with permission from [103], copyright 2013 The Royal Society of Chemistry.

In 2011, Sun et al. showed first results on an aprotic, room temperature sodium oxygen cell (Figure 10a) [101]. In contrast to Peled et al. they made use of a solid sodium foil as anode and a diamond-like carbon thin film electrode as cathode. In accordance with typical lithium–oxygen cells they used 1 M NaPF_6_ in EC:DMC 1:1 as the liquid, aprotic electrolyte. The cell setup was an H-shaped glass cell. Using transmission electron microscopy, single area electron diffraction and Fourier transform infrared spectroscopy sodium peroxide (Na_2_O_2_) and sodium carbonate (Na_2_CO_3_) were proven as discharge products. These products vanished during charge with overpotentials exceeding 1 V similar to lithium–oxygen cells. Overall, the cell performed just like a typical lithium–oxygen battery, however, the discharge potentials were slightly lower (around 2.4 V), as expected. In 2013, the same group (Liu et al., [103]) used graphene nanosheets as cathode and NaPF_6_ dissolved in monoglyme as electrolyte. This way, discharge capacities as high as 9268 mAh/g_carbon_ were achieved. Again, sodium peroxide was described as the discharge product and large overpotentials were observed (Figure 10b). In both cases, the voltage profile can be classified as Type 2C.

**Figure 11:**
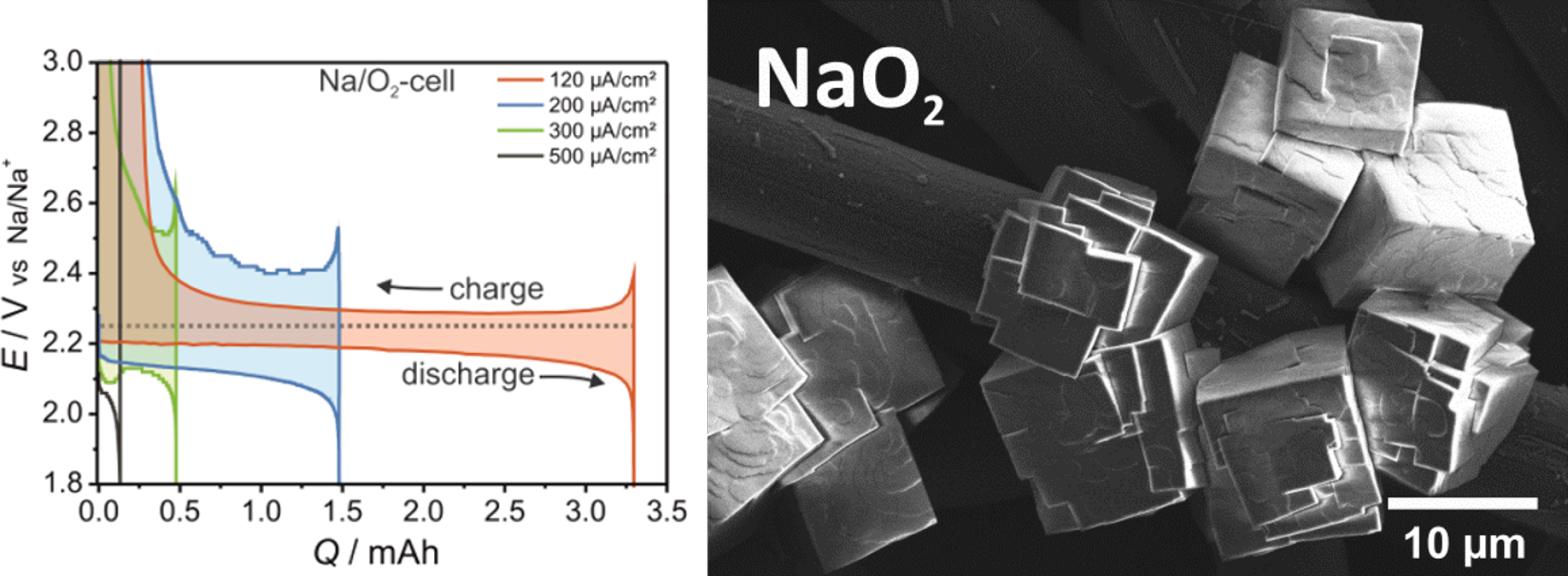
Discharge/charge curves (Type 1B) of a sodium–oxygen battery with NaO_2_ as discharge product. The main differences compared to Li/O_2_ cells are that only small overpotentials are observed and that the crystallite size of the discharge product is much larger (see SEM image on the right) [109].

In 2012 Hartmann et al. [109] reported a sodium–oxygen battery with sodium superoxide (NaO_2_) as discharge product. Unequivocal proofs for superoxide formation were provided by X-ray diffraction, Raman spectroscopy and pressure monitoring. SEM studies revealed that, in contrast to Li/O_2_ cells for which nanoscopic Li_2_O_2_ toroids are found, NaO_2_ forms large micrometer-sized cubic crystallites (compare Figure 7 with Figure 11). The cells showed only very small combined overpotentials of about 200 mV during cycling which was attributed to the kinetically favored one-electron transfer. Shortly after, similar findings were reported for potassium–oxygen cells. Here, KO_2_ forms during discharge and a very similar voltage profile has been found [20]. The Coulombic efficiency of the sodium superoxide cell in the first cycle was around 90%, discharging and charging ended with a sudden voltage drop and increase, respectively. The voltage profile can therefore be classified as Type 1B, meaning that the cell cycles more ideal than Li/O_2_ cells or Na/O_2_ cells with peroxides as discharge products. The achieved discharge capacity with 300 mAh/g_carbon_ was relatively low due to the high mass of the free standing electrode. On the other hand, the absolute capacities were comparably high. Cycle life, however, was poor and the capacity faded to virtually zero within ten cycles. The study also included a direct comparison in cycling behavior between otherwise identical Na/O_2_ and Li/O_2_ cells. The latter showed a much smaller discharge capacity and the expected large overpotentials. Although the Na/O_2_ cell with NaO_2_ as discharge product shows a much more reversible cell reaction compared to the Li/O_2_ cell, it should be noted that also the Na/O_2_ cell is not entirely free from side reactions either. Overall, this study provided clear evidence that lithium–oxygen and sodium–oxygen batteries can behave completely different.

Later on, the same group published a more comprehensive study on their findings using a range of different methods including DEMS, pressure monitoring, XPS, SEM, UV–vis spectroscopy, XRD and Raman spectroscopy [78]. The reason why NaO_2_ grows to such large crystals is still not clear yet, but precipitation of NaO_2_ from a supersaturated solution was suggested as a possible growth mechanism. XPS studies showed that the reason for the poor overall reversibility might be due to decomposition of the conductive salt. Further, the issue of dendrite formation in Na/O_2_ cells was discussed.

Kim et al. studied the influence of the electrolyte solvent on the discharge product in sodium–oxygen cells [106]. The electrode was made of Ketjenblack, a typical high surface area carbon. Capacities of 2800 mAh/g and even 6000 mAh/g were reported for PC and tetraglyme, respectively. The voltage profiles were of Type 2C. The discharge product was not the same as reported in literature before. Using FTIR spectroscopy and X-ray diffraction they found that sodium carbonate was the major discharge product for carbonate based electrolytes and hydrated sodium peroxide (Na_2_O_2_·2H_2_O) was the discharge product for tetraglyme. The authors suggested that the water molecules stem from the irreversible decomposition of the electrolyte. But comparing this result to the study by Hartmann et al. who found NaO_2_ using diglyme as solvent, it becomes clear that a direct link between ether solvents and formation of Na_2_O_2_·2H_2_O cannot be drawn. Indeed, the reason why different groups find different discharge products is not clear yet.

Liu et al. studied the influence of nitrogen doping of the carbon electrode on the performance of sodium–oxygen batteries [103]. Compared to a pure graphene cathode the doped one showed considerably higher discharge capacities reaching up to 8600 mAh/g_carbon_. In both cases, Na_2_O_2_ formed during discharge as evidenced by XRD. Galvanostatic cycling and cyclic voltammetry revealed that nitrogen doping is effective in reducing the overpotentials during discharge and charge. The hysteresis, however, can be still classified as a Type 3B. SEM was used to study the morphology of the discharge product as a function of the discharge current. In line with what is known from Li/O_2_ cells, particles form at low currents whereas film formation is observed at higher currents.

Only a short time later another high capacity cathode was presented by Jian et al. [107]. They used a carbon nanotube electrode in combination with two different electrolytes, namely NaTFSI in tetraglyme and NaTfO in diglyme. Although the latter showed a higher discharge capacity (7530 mAh/g compared to 6000 mAh/g), the overall performance was similar. During discharge hydrated sodium peroxide was formed as evidenced by XRD. Charging started at small overpotentials but was quickly followed by a rapid increase in voltage. Only 50% of the capacity could be recovered during charging. The performance could be improved by shallow cycling at around 13% of the full capacity, however, all voltage profiles can by classified as Type 3B.

Additional physicochemical aspects of the Na/O_2_ cell with NaO_2_ as discharge product were discussed by Hartmann et al. in 2014 [110]. Here, pressure monitoring was successfully combined with the standard electrochemical methods galvanostatic cycling and cyclic voltammetry. Furthermore, electrochemical pressure impedance spectroscopy (EPIS) was introduced as a tool to study the transport properties within the cell. With this, the experimental data were fitted by a quantitative microkinetic model that is based relevant parameters and transport process describing the cell. Further, solubility and diffusion coefficients of oxygen in several solvents were determined and operation of the Na/O_2_ cell under mixed O_2_/N_2_ gas atmosphere was demonstrated. Importantly, NaO_2_ was found as discharge product despite the addition of nitrogen gas. On the other hand, the discharge capacity under synthetic air was much lower compared to pure oxygen. This result underlines that metal–air batteries need to be studied also at lower oxygen partial pressures when aiming at practical applications.

Around the same time two theoretical studies were published. Lee et al. studied the phase stabilities of different possible discharge products as a function of the oxygen partial pressure and calculated that NaO_2_ and respectively Li_2_O_2_ are most stable under standard conditions [115]. Surface energies were calculated and used to predict the Wulff equilibrium shape of the different phases. The cubic crystallites predicted for NaO_2_ are well in line with what has been experimentally reported (see Figure 11). Finally, it was calculated that the OER from superoxides is kinetically favored compared to peroxides. Kang et al. studied the phase stabilities of sodium–oxygen compounds as a function of temperature, partial pressure and, importantly, also crystal size [116]. In contrast to the results of Lee et al., they found that Na_2_O_2_ is the most stable phase at standard conditions in the bulk phase. In the nanometer regime, however, NaO_2_ becomes more stable due to its lower surface energy. The threshold under standard conditions is approximately reached for crystal sizes of ≈6 nm in diameter. For the same reason, also nucleation of NaO_2_ is preferred over Na_2_O_2_ at any oxygen pressure and temperature. The authors state that NaO_2_, once nucleated during discharge, may never transform to Na_2_O_2_.

The fundamental difference in cell behavior between otherwise identical Li/O_2_ and Na/O_2_ cells was further pointed out by McCloskey et al. [67]. They compared lithium–oxygen to sodium–oxygen cells with ether based electrolytes by means of DEMS measurements. Ratios for *n*(e^–^)/*n*(O_2_) of around 2 and 1 were found for the different cells, respectively, indicating formation of Li_2_O_2_ in Li/O_2_ cells and formation of NaO_2_ in Na/O_2_ cells. In line with other studies finding NaO_2_, the voltage hysteresis showed a Type 1B behavior, that is, small overpotentials during charging (≈200 mV) and a sudden voltage increase at the very end of charging. The Li/O_2_ cell showed Type 3C behavior, that is, an increase in voltage during charging resulting in very high overpotentials of more than 1.5 V. Interestingly, this significant difference in overpotentials is not seen by cyclic voltammetry using a glassy carbon working electrode. The authors suggest that the difference in overpotentials between lithium and sodium based cells is due to the different reactivity of the discharge products: During charging, Li_2_O_2_ reacts with the electrolyte and carbon cathode to form Li_2_CO_3_ leading to a continuous increase in overpotential. In contrast, NaO_2_ is less reactive and hence no Na_2_CO_3_ forms. As a consequence, overpotentials during charging remain small.

**Figure 12:**
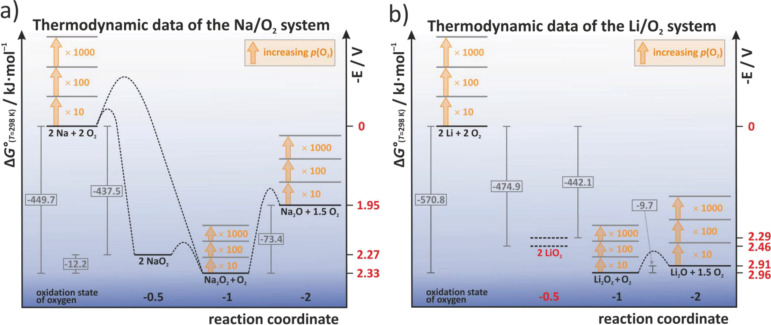
The thermodynamic landscape of (a) sodium– and (b) lithium–oxygen cells. All values are calculated for the reaction 2A + 2O_2_ → A_2_O_y_ + (2–y/2)O_2_, where y = 1,2,4. Figure adapted with permission from [27], copyright 2014 Wiley-VCH.

Bender et al. discussed possible origins for the different discharge products observed in Li/O_2_ and Na/O_2_ cells by comparing tabulated thermodynamic data of the different phases [27]. A graphical representation is shown in Figure 12 and is based on thermodynamic data of the bulk phases (*T* = 298 K, *p* = 1 bar). The kinetic barriers shown are only a guide to the eye as absolute values are not known. Three relevant aspects can be seen: (1) In both systems, the peroxide is thermodynamically most stable at standard pressure and should therefore form as discharge product, (2) In the Na/O_2_ system, NaO_2_ and Na_2_O_2_ are thermodynamically quite close, whereas in the Li/O_2_ system, Li_2_O_2_ and Li_2_O are very close. For Na/O_2_ cells this means that the cell voltages for NaO_2_ (2.27 V) and Na_2_O_2_ (2.33 V) formation are very close. Given the uncertainty of the thermodynamic data it becomes clear that the discharge mechanism cannot be simply derived from the discharge potential. (3) The phase stability naturally depends on the oxygen partial pressure, meaning that NaO_2_ or LiO_2_ might become more stable than the peroxides at elevated pressures. For NaO_2_, the threshold can be estimated to 133 bar, which well explains why the chemical synthesis of phase pure NaO_2_ from Na_2_O_2_ in autoclaves occurs at partial pressures and temperatures of around 280 bar and 475 °C [117].

**Figure 13:**
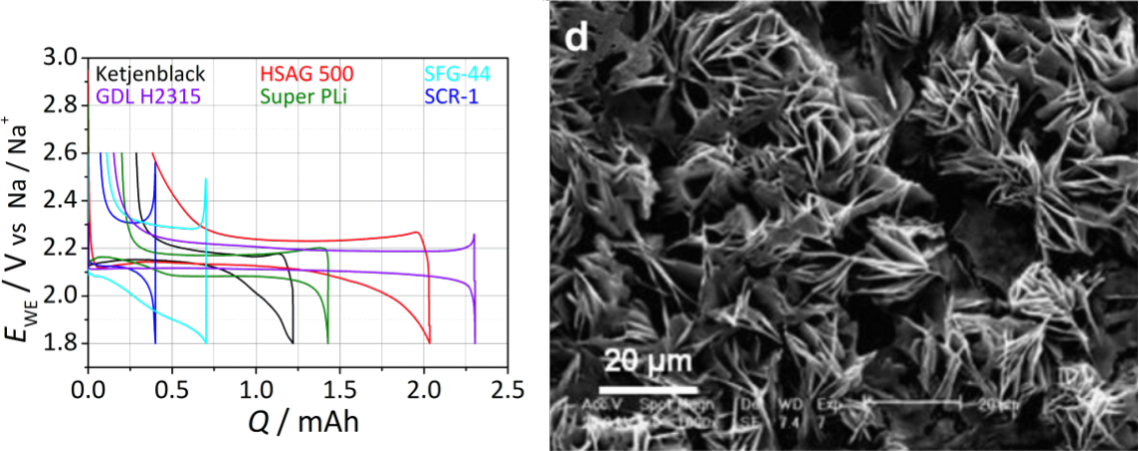
Voltage hysteresis of different carbon materials for the cathode of a sodium oxygen cell (left), figure adapted with permission from [27], copyright 2014 Wiley-VCH. SEM image of the oxygen electrode of a Na/O_2_ cell after discharge with Na_2_O_2_ and Na_2_CO_3_ as discharge product (right), figure adapted with permission from [105], copyright 2014 Elsevier.

The authors suggested that as the energetic difference between NaO_2_ and Na_2_O_2_ is so small (about 12 kJ/mol), slight differences in the kinetic properties might lead to either of them as discharge products. A reasonable assumption for what controls the kinetics of the cell reaction is the type of carbon electrode. Indeed, the different groups reporting on Na/O_2_ cells all used different carbon materials which might explain the different findings. The authors therefore tested a range of different carbon materials but concluded that the type of carbon has no influence on the nature of the discharge product as in all cases NaO_2_ was found as major discharge product. Overall, Type 1B behavior was found in all cases. The achievable capacities, however, were significantly affected by the type of carbon (Figure 13, left). Furthermore, shallow cycling at around 33% of full capacity enabled cycling of the cell for more than 50 cycles with a capacity of 1666 mAh/g using a Ketjenblack electrode with 0.5 M NaOTf in diglyme as electrolyte.

Liu et al. substituted the commonly used carbon electrode by a nickel based composite electrode consisting of nickel foam covered with NiCo_2_O_4_ nanosheets [105]. NaClO_4_ in monoglyme was used as electrolyte. The pure nickel foam was shown to be inactive. For the composite, however, a discharge capacity of 1762 mAh/g (at 20 mA/g based on the mass of the nanosheets was found). A strong capacity fade was observed during cycling. The voltage profiles can be classified as Type 3B/3C. IR spectroscopy and TEM/SAED were used to determine the discharge products. Sodium peroxide and, as a result of side reactions, Na_2_CO_3_ were found. The electrodes after discharge were further studied by SEM. Flat sheets with a diameter of around 20 µm were found (Figure 13, right). Obviously, this morphology is very different from the cubic particles reported for cells with NaO_2_ formation.

**Figure 14:**
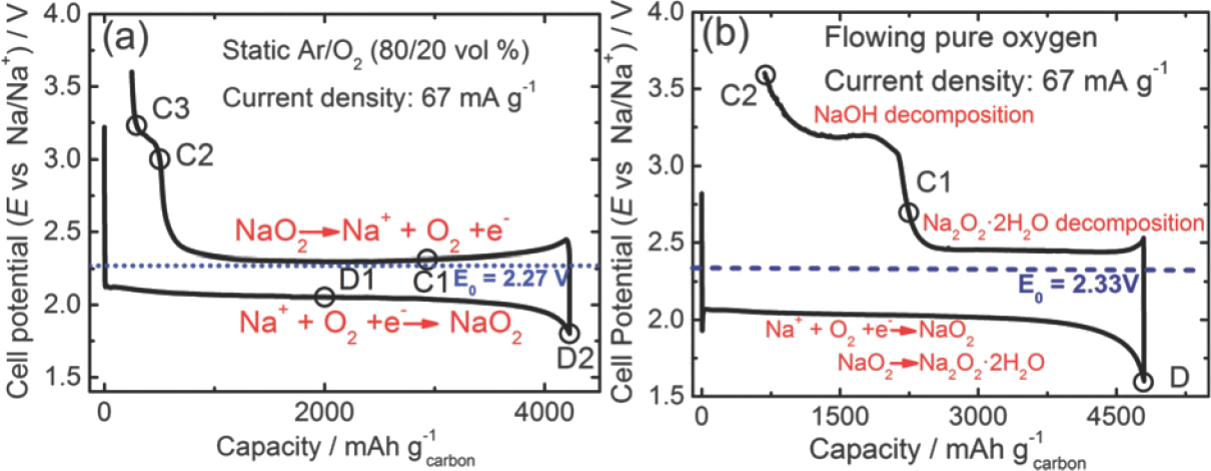
Voltage profiles of Na/O_2_ cells under static gas atmosphere and flowing gas atmosphere (Type 1B/3B). Figure adapted with permission from [111], copyright 2014 The Royal Society of Chemistry.

Another study discussing reasons for the different types of discharge products reported in literature was published Zhao et al. [111]. Vertically aligned carbon nanotubes grown on a steel substrate were used as oxygen electrode, sodium triflate in tetraglyme was used as electrolyte. Voltage profiles were of Type 1B and consequently also NaO_2_ in form of cubic particles was observed as discharge product. The cell delivered a capacity of more than 4000 mAh/g_carbon_. Improved cycle life was achieved with shallow cycling at 750 mAh/g (19% DOD). More than 100 cycles have been achieved this way. Rate performance was improved by electrochemically predepositing a thin layer of NaO_2_ at low currents (67 mA/g). This procedure was applied to increase the overall number of nucleation sites for product formation during subsequent cycles at higher currents. By doing so, a capacity of around 1500 mAh/g was achieved at 667 mA/g, for example. An important feature of the study was that the cells were not only cycled under static atmosphere in a sealed container but additionally also under continuous gas flow. Pure oxygen or an Ar/O_2_ (80/20) mixture were used. Interestingly, the authors found NaO_2_ under static conditions and Na_2_O_2_·2H_2_O under continuous gas flow. The authors suggest that humidity is likely to be introduced when applying a constant flow (presumably due to leakage or gas impurity). Charging was followed by XRD and it was found that Na_2_O_2_·2H_2_O decomposes to form water, O_2_ and NaOH leading to higher overall potentials and a Type 3B behavior, see Figure 14. It is important to note that a continuous gas flow is closer to the operation mode of a practical cell operating with atmospheric oxygen. Further studies are therefore needed to clarify the source and impact of H_2_O on the cell reaction.

Yadegari et al. studied the relation between specific surface area and discharge capacity using chemical activation of commercial carbon black by NH_3_ or a CO_2_ gas [108]. Sodium triflate in diglyme was used as electrolyte. The results can be summarized as follows: The longer the chemical treatment, the higher the specific surface area, the higher the discharge capacity. The major discharge product was Na_2_O_2_·2H_2_O although small amounts of Na_2_O_2_ and NaO_2_ were also detected by combining different methods. As the PVDF binder used in this study is known to be unstable against the superoxide radical, the authors suggested that the formation of the hydrated peroxide is related to the binder decomposition. As a result of the complex mixture of discharge products, the charging curves were characterized by several steps. Overall, all voltage profiles were of Type 3C. The morphology of the electrode after discharge showed quite some similarities compared to the study by Liu et al. It was further shown that the discharge rate influences the voltage behavior during charging.

#### Overall comparison

**Figure 15:**
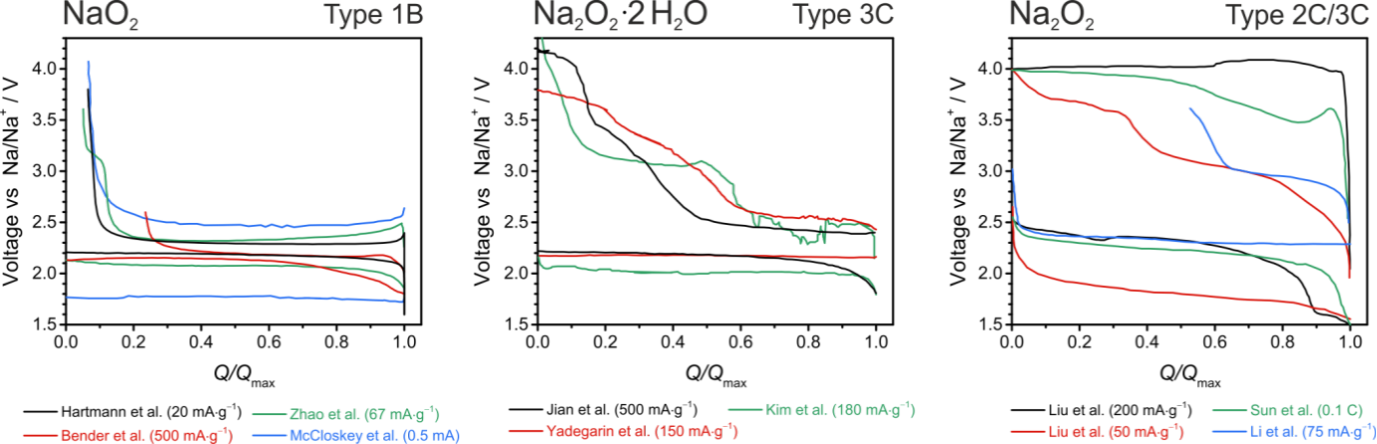
Literature overview on different studies of Na/O_2_ cells. The comparison shows the voltage profile of the first cycle. Data has been digitalized from the different publications. Only cells with NaO_2_ as discharge product show a defined voltage hysteresis, combined with low overpotentials and a defined end point during recharge. Please note that some groups measure in 3-electrode, others in 2-electrode configuration.

For a better comparison of the published literature, we digitalized the voltage profiles and grouped them according the different discharge products. The result is shown in Figure 15. Groups finding sodium superoxide as discharge product find a Type 1B behavior with low overpotentials and a sudden voltage increase once the end or recharge is reached. Efficiencies are typically above 80%. Groups finding Na_2_O_2_·2H_2_O as discharge product find a Type 3C behavior. Characteristic for this behavior are increasing potentials and no defined end point of charge, indicating a complex charging mechanism and side reactions. Different sources for H_2_O have been suggested, but its origin is still a matter of debate. Groups finding Na_2_O_2_ as discharge product usually observe voltage profiles with Type 2C or 3C behavior. A sudden or sloping increase in potential during charging and no defined end point of charge are observed in these cases.

#### Related concepts

In addition to the studies discussed so far some other related concepts have been suggested. Das et al. proposed a cell concept that mainly aims at CO_2_ capture while at the same time generating electrical energy [102]. Their cells can be therefore described as Na/(O_2_ + CO_2_). The authors investigated the cell discharge behavior under different gas ratios and found that a 50:50 mixture of O_2_ and CO_2_ yielded higher discharge capacities than the single gases. Na_2_CO_3_ and Na_2_C_2_O_4_ were suggested as discharge products. No charging curves were shown as the cell was designed as primary cell. In a later study, the same group used an organic/inorganic hybrid liquid electrolyte in order to enable partial recharge [118]. The voltage profiles are of Type 3C and show combined overpotentials of up to around 2.5 V. The discharge product was found to be NaHCO_3_.

Hayashi et al. published results on a Na/O_2_ battery with a mixed aqueous/aprotic electrolyte. Both electrolytes were separated by a Nasicon solid electrolyte [119]. Discharge capacities of about 600 mAh/g (based on the weight of Na and H_2_O) with NaOH as the discharge product were achieved, which is only 30% lower than the theoretical capacity of the cell reaction; however, no data on rechargeability was shown. The concept of combining different types of electrolytes has been already applied for Li/O_2_ cells. But the authors point out that the much higher solubility of NaOH in aqueous electrolytes compared to LiOH might be of an important advantage. Clogging of the cathode by precipitated hydroxide might be delayed and an even higher energy density could be obtained.

### 3 Lithium–sulfur (Li/S_8_) and sodium–sulfur (Na/S_8_) batteries

#### 3.1 Operating principles and general remarks

**Figure 16:**
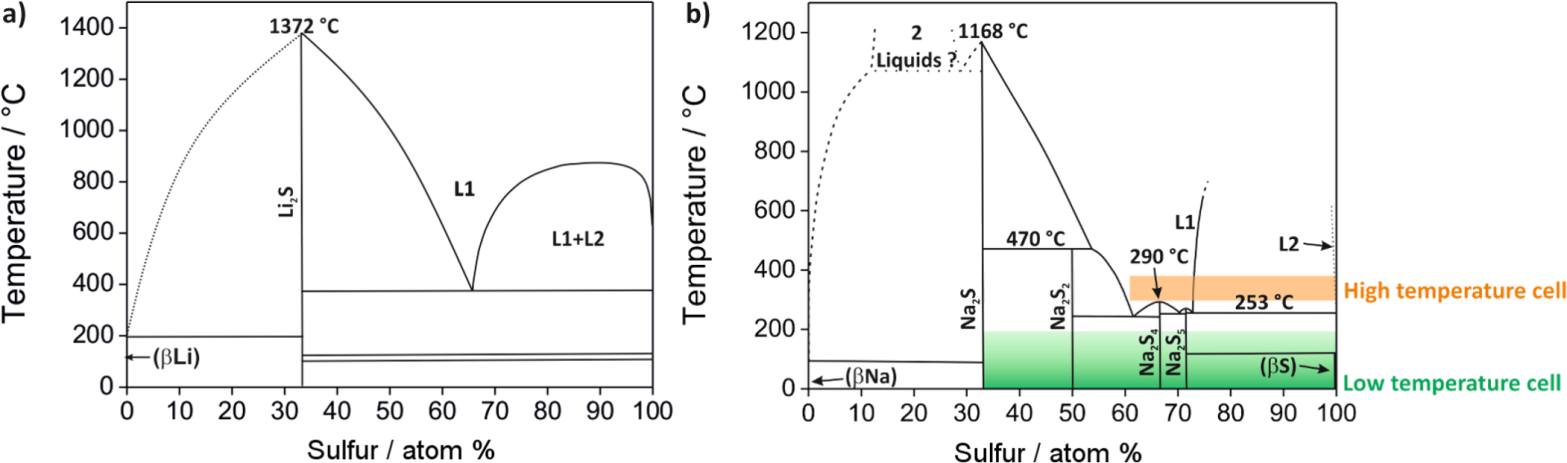
(a) The Li–S phase diagram. (b) The Na–S phase diagram. Redrawn from references [129–130]. The Na–S phase diagram also depicts the operating window of the commercialized high temperature cell and alternative cell concepts operating at low temperature – including room temperature – that are on the research level.

**Figure 17:**
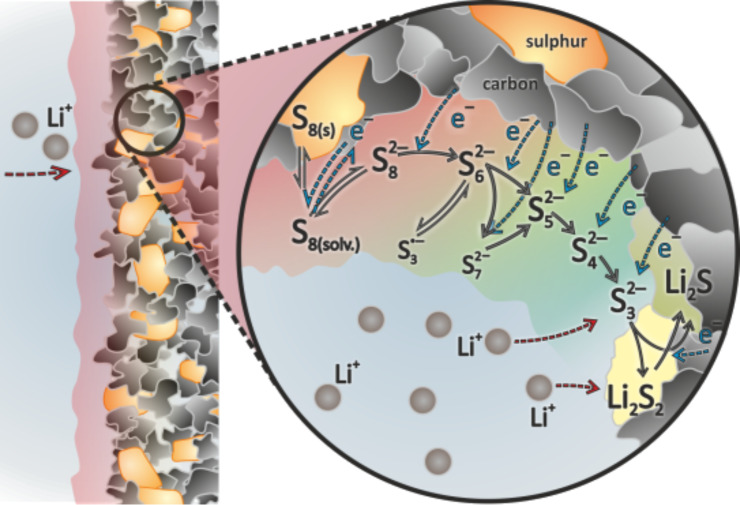
Schematic illustration of the reduction processes at the negative electrode during discharge of a Li/S_8_ battery. Reduction of sulfur S_8_ proceeds over several soluble polysulfide intermediates (Li_2_S*_x_*) before the final precipitation of solid phases, Li_2_S and eventually Li_2_S_2_ occurs. The cell discharge can be also followed by UV–vis spectroscopy, as different polysulfides give rise to different coloration. Illustration adapted from [124].

**Figure 18:**
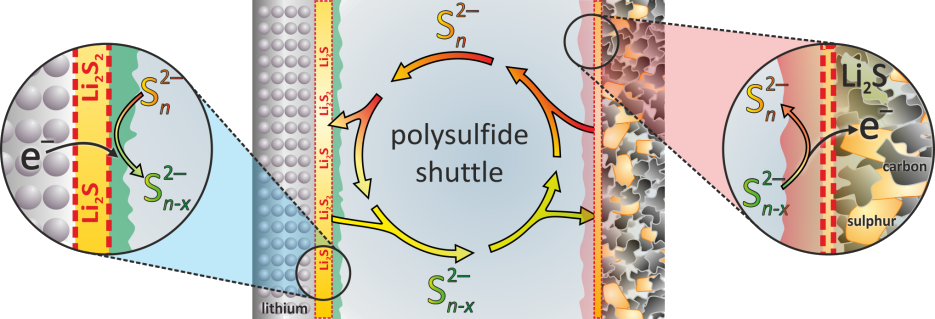
Schematic illustration of the polysulfide shuttle mechanism after Mikhaylik and Akridge [123]. Long polysulfides diffuse towards the lithium electrode where they are reduced to shorter polysulfides. Subsequently, these shorter polysulfides diffuse back to the positive electrode where they are oxidized. As a result, a cyclic process (“shuttle mechanism”) develops that corresponds to a chemical shortcut of the cell. Illustration adapted from [124].

The lithium–sulfur battery system has been studied for several decades. The first patents and reports on lithium–sulfur batteries date back to the 1960s and 70s [120–122]. However, a rapid increase in research efforts and progress in development was only achieved within the last 10 to 15 years. The number of research publications is growing exponentially. The most studied cell concept is based on lithium as a negative electrode and solid sulfur as a positive electrode. Lithium sulfide (Li_2_S) is the final discharge product and the only thermodynamically stable binary Li–S phase, as shown in Figure 16a. The theoretical cell voltage of 2.24 V is comparably low but due to the high capacity of sulfur (1672 mAh/g) the theoretical energy density by weight (2615 Wh/kg) exceeds that of LIB by a factor of five. The basic cell concept of a lithium–sulfur battery is depicted in Figure 2c. The main challenges of the lithium–sulfur battery are related to two intrinsic properties:

1. Sulfur and Li_2_S are insulators, and intimate contact to a conductive support and sufficiently small particle sizes are necessary to render a complete cell reaction. At the same time, the support must accommodate the volume change of 80% that arises from the difference in molar volumes of sulfur (15.5 mL/mol) and Li_2_S (28.0 mL/mol).
2. Formation of Li_2_S from sulfur does not occur directly but via a series of polysulﬁde intermediates (Li_2_S_2_ and Li_2_S*_x_*, *x* > 2). Polysulﬁdes of the stoichiometry Li_2_S*_x_* are highly soluble in commonly used electrolytes, meaning that the active material diffuses out of the positive electrode and eventually reacts with the negative electrode or deposits somewhere else in the cell where it remains inactive. So cycling sulfur in a Li/S_8_ battery is essentially based on dissolution and precipitation processes as schematically illustrated in Figure 17. Despite several efforts, however, it is still not well understood in which amounts and stoichiometries polysulfides form. The polysulﬁde solubility leads to a parasitic phenomenon called the ‘‘shuttle mechanism’’ [123] (Figure 18) that corresponds to a chemical shortcut of the cell. This effect essentially leads to continuous self-discharging during discharge, charge and rest. The degree of the shuttle effect heavily depends on the experimental conditions. Shuttling becomes stronger at small current and/or higher temperatures [123–124]. Moreover, also sulfur S_8_ itself is mobile and was found to diffuse rapidly [125].

**Figure 19:**
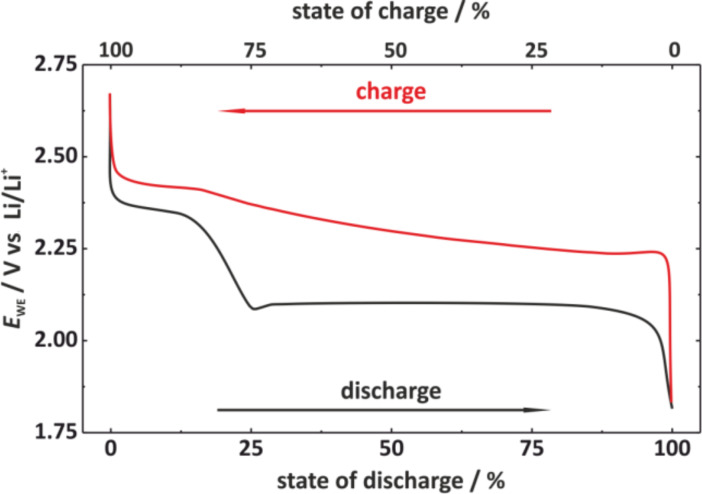
Typical voltage profile of a lithium/sulfur cell. A similar behavior can be expected for an analogous sodium/sulfur cell.

The complex cell reaction gives rise to a characteristic discharge/charge profile as shown in Figure 19. Both the discharge and the charge voltage profiles consist of two voltage plateaus occurring at about 2.3 V and 2.1 V (discharge) or 2.3 V and 2.4 V (charge), respectively. Within the higher discharge plateau the soluble intermediate polysulfides are formed, corresponding to reduction of S^0^ to S^−0.5^ (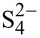), accounting for a quarter of the overall capacity. Further reduction leads to formation and precipitation of insoluble species leading to an overall two electron reduction of S with Li_2_S as end product. During the following charge, Li_2_S is reconverted to S_8_ via intermediate polysulfides, ideally_._ The characteristic minimum between the upper and the lower discharge plateau is attributed to the nucleation of solid products [126–127]. The exact position of the potentials also depends on the electrolyte solvent [128].

As a result of these effects, the Coulombic efficiency is low, utilization of sulfur in Li/S_8_ cells is poor and the capacity diminishes within a few cycles. Therefore special measures have to be taken in order to improve the performance of Li/S_8_ cells.

The most frequently applied strategy to improve the cell performance is to use (nano)porous carbon materials as support that provide high surface area and electronic conductivity and at the same time prevent or delay the loss of active material towards the electrolyte. Electrode mixtures are prepared by simply mixing the carbon materials with sulfur or by infiltrating the carbon matrix with molten sulfur above its melting point (*T*_m_ = 119 °C). A typical electrode for Li/S_8_ batteries then contains typically around 50–70 wt % sulfur, 30–50 wt % carbon and a small amount of binder. For comparison, the amount of carbon as conductive additive for electrodes in conventional LIBs is well below 5 wt %.

During the last 5–10 years, a large number of different sulfur/carbon nanocomposite materials has been studied and often considerable improvements in terms of sulfur utilization and cycle life were achieved compared to cells with conventional carbon materials. Overall, nowadays several tenths to several hundreds of cycles with capacity values around 700–1000 mAh/g are realized and the combined overpotentials in the first cycles are roughly around 200 mV. But whether the improvements are really due to specific structural properties of the nanocomposite is, however, not easy to answer considering the complexity of the possible reactions in a lithium–sulfur cell. It also turned out that the characterization of sulfur/carbon nanocomposite materials may pose problems and results can be misleading due to the high sulfur mobility [125]. The main issue, however, is that the performance of Li/S_8_ cells is particularly sensitive to the properties of the electrode (thickness, sulfur content, sulfur loading, preparation method, etc.) and the amount of electrolyte and lithium. In fact, quite reasonable results can be obtained with commercially available carbon materials once the electrode preparation is optimized [131–132]. Assessing the achievements of the last years, in general, long cycle life and high sulfur utilization has so far obtained only for low sulfur loadings (often <1 mg/cm^2^) and large excess of both electrolyte and lithium. Excess of lithium and electrolyte are necessary as both continuously react with each other during cycling. However, low loadings and large excess of lithium and electrolyte are no option for practical devices, and it will be the key to competitive Li/S_8_ cells to bring cathodes with high sulfur loading (about 5 mg/cm^2^) and a low electrolyte/sulfur ratio to function [131,133–137]. Overall, to enable a high energy battery, the electrolyte:sulfur ratio should be smaller than 5:1 (for comparison, the ratio of electrolyte and active material in conventional LIBs is around 1:3) and the sulfur content of the electrode should be at least 70% providing at least 2–4 mAh/cm^2^ (i.e., the typical areal capacity for LIBs).

Besides the attempts to improve the cathode design, also a number of other strategies are followed in order to improve the performance of lithium/sulfur batteries (see section, The lithium–sulfur (Li/S_8_) battery). The cell concept shown in Figure 2c is by far the most studied one but also other concepts have been proposed. The high solubility of polysulfides can be used to design cells with a liquid electrode (catholyte), for example. Although this concept has been studied already many years ago [121], it only recently regained attention [138]. On the other hand, solid-state concepts are being considered [139–141].

The theoretical energy densities of the lithium–sulfur battery are summarized in Table 3. But from the above arguments it becomes clear that experimental energy densities will be much lower. No lithium–sulfur cell has been commercialized yet but several companies announced (gravimetric) energy densities for rechargeable cells significantly exceeding lithium-ion technology. Sion Power currently reports 350 Wh/kg on the cell level but aims for over 600 Wh/kg and 600 Wh/L in the near future [142]. Oxis Energy reports 300 Wh/kg (2014) and predicts 400 Wh/kg (forecast in 2016) [143]. The rate capability of lithium–sulfur cells is thought to be competitive with high-rate LIBs [144]. At moderate rates of C/10, the combined overpotentials of Li/S_8_ amount to roughly 150–250 mV. By and large, the lithium–sulfur cell as rechargeable energy store appears to have a realistic chance for commercialization, but will compete with continuously optimized LIB.

**Table 3:** Theoretical cell voltages, gravimetric and volumetric energy (Wh/kg, Wh/L) and charge (mAh/g, mAh/cm^3^) densities for lithium– and sodium–sulfur batteries with a metal anode. Due to the large differences in their densities, the volumetric energy densities of metal–sulfur cells strongly depend on whether they are in the charged or discharged state. Charge densities refer to the discharged state, that is, to the sulfides. Thermodynamic data were derived from HSC Chemistry for all compounds in their standard state at 25 °C or 300 °C. Densities at 300 °C are estimates. In contrast to LIBs, metal–sulfur cells are usually assembled in the charged state. The theoretical capacity of the positive electrode is therefore usually given based on the mass of sulfur only, so the theoretical capacity is *Q*_th_ = 1672 mAh/g for full reduction of sulfur to form Li_2_S or Na_2_S.

Cell reaction	*E*° / V	*W*_th_ / Wh/kg	*Q*_th_ / mAh/g	*W*_th_ / Wh/L	*Q*_th_ / mAh/cm^3^

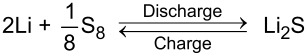	2.24	2615	1167	4289 / 2896	1914
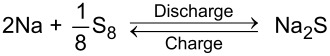	1.85	1273	687	2364 / 1580	1245
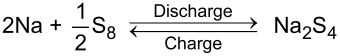 (25 °C)	2.03	626	308	1326 / 997	653
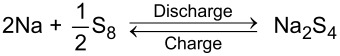 (300 °C)	1.90	583	308	1124 / 845	653
Li-ion (average cathode vs Li/Li^+^)	3.8	530	140	2300	600

In contrast to the lithium–sulfur battery, the analogue room temperature sodium–sulfur battery has been hardly studied to date but the challenges for the construction of well functioning cells will be quite similar. However, the theoretical energy density of a Na/S_8_ cell is roughly 50% smaller compared to the analogous Li/S_8_ cell, due to higher atomic mass of sodium. So if only energy density is considered, the Na/S_8_ cell will not be competitive with LIB technology both in terms of volumetric and probably also gravimetric energy density. Besides, the even larger volume change of the sulfur electrode during cycling (170% for Na_2_S formation compared to 80% for Li_2_S formation) will pose additional problems.

A look at the phase diagrams shows that different cell reactions might occur in Li/S_8_ and Na/S_8_ cells, as several Na_2_S*_x_* compounds are thermodynamically stable at room temperature. This means that during cell discharge, polysulfides might not only dissolve in the electrolyte, but may also precipitate as solids. Whether the stability of solid Na_2_S*_x_* polysulfides is of advantage or disadvantage for a reversible cell reaction remains an open question, but – generally speaking – solid phases are likely to have detrimental effects on the cell kinetics compared to dissolved Na_2_S*_x_* species. It is worth noting that also Na_2_S_3_ has been reported as stable phase, however, it turned out to be a eutectic mixture of the stable polysulfides Na_2_S_2_ and Na_2_S_4_ [130]. The Na–S phase diagram (see Figure 16b) also depicts the high-temperature Na/S_8_ cell that operates with molten electrodes and a solid electrolyte. As the polysulfides Na_2_S*_x_* have high melting points, the cell reaction at around 300 °C is limited to a narrower stoichiometric window, meaning that full reduction of sulfur cannot be achieved. The theoretical energy density for high temperature Na/S_8_ cells is therefore limited. In practice, 200 Wh/kg has been achieved on the battery level.

Overall, one can look at the room-temperature Na/S_8_ cell from two perspectives: (1) Compared to a Li/S_8_ cell, substituting lithium by the more abundant sodium appears attractive, and the same strategies for improving Li/S_8_ batteries (sulfur utilization, cycle life) might apply for Na/S_8_ batteries. An advantage for sodium could be that sodium solid electrolytes are commercially available, that would enable efficient protection of the metal anode from polysulfides. On the other hand, the theoretical energy densities are lower and the larger volume expansion might lead to severe problems. (2) Compared to a high-temperature Na/S_8_ cell, decreasing the operating temperature would be attractive because safety and corrosion issues are reduced. In addition, if full reduction of sulfur to Na_2_S can be accomplished, an increase in system's energy density might be possible.

A compromise could be to operate the cell at intermediate temperatures below 200 °C [145–147]. Here, the sodium anode (*T*_m_ = 98 °C) can be either solid or liquid, a NASICON-membrane (Na Super Ionic Conductor) or beta-alumina membrane is used as solid electrolyte and the cathode is based on a mixture of sulfur or Na_2_S*_x_* in an organic solvent. Such an approach has been already discussed in 1980 by G. Weddigen [148].

#### 3.2 State-of-the-art and recent developments

**3.2.1 The lithium–sulfur (Li/S_8_) battery:** As mentioned earlier, a considerable number of papers are currently being published in the field of lithium–sulfur batteries. This summary is intended to highlight the key strategies currently followed for improving the performance of Li/S_8_ batteries. The same strategies might be adopted to improve the performance of the analogue room temperature Na/S_8_ battery, although research in this field is still on an exploratory level. For a more comprehensive and complete overview on lithium–sulfur batteries, the authors refer to more specialized reviews [149–155].

The challenges of the Li/S_8_ system address all of its main components. Hence, main approaches striving to find a solution for these challenges, address (1) cathode composition and architecture, (2) electrolyte composition and additives and (3) improvements or alternatives to the Li anode. Beyond the improvement of the single components – both from fundamental and engineering point of view – a comprehensive understanding of the complicated redox chemistry of the Li/S_8_ system has to be obtained. Therefore, the demand in analytics and simulation studies of the electrochemistry is constantly growing. This section will close with an outlook to new cell design approaches to address the special chemistry of Li/S_8_ batteries.

3.2.1.1 Cathode: The ideal cathode of a lithium–sulfur battery should provide the following features: (a) A high electronic conductivity and fine dispersion of the active material to achieve a complete active mass utilization and high rate capability. (b) A structure confining the active mass to prevent the loss of polysulfides and hence the shuttle effect. (c) A flexible structure to accommodate the volume changes during cycling. (d) A sufficient active mass loading to compete at least with current lithium ion batteries (LIBs). Points a–c can be addressed by developing and engineering conductive supports. Mostly porous carbon or carbon composite materials are used for this purpose. Again, we emphasize that the sulfur loading on the electrodes needs to be sufficiently high in order to achieve high energy densities in practice. For example, a sulfur loading of more than 2 mg/cm^2^ and 100% sulfur utilization is necessary in order to reach technically relevant areal capacities of about 3.5 mAh/cm^2^. This aspect has been often overlooked in the last years but needs to be considered when claims on the practical rather than the academic relevance of new electrode architectures are made.

A few of the recent approaches are highlighted in the following. General remarks on the electrode preparation methods will be given at first.

Electrode preparation and binders: Intimate contact between carbon and sulfur is usually obtained by heating sulfur/carbon mixtures above the melting point of sulfur, leading to melt infiltration of the porous support. Some more specific approaches combine a first melting step followed by evaporation of excess surface–sulfur [156] or deposition of sulfur over the gas phase [157]. Apart from some binder-free cathode approaches (see below), binders play a particularly important role when preparing the final electrodes from the sulfur/carbon mixtures. Beyond the ability to bond the cathode components and link them to the current collector, binders have to be flexible enough to accommodate the volume change. Furthermore, they should favor a maximum dispersion of the active material and the conductive agent and limit polysulfide dissolution. Established binders for LIBs such as polytetrafluorethylene (PTFE) or polyvinylidene fluoride (PVDF) have been used long time for Li/S_8_ cells but may not provide sufficiently good properties. Polyethylene glycol (PEO, PEG) as one of the earliest alternative binders may improve cycle life [158–159] by electrolyte modification through partial dissolution. As first published by Sun et al. [160], gelatin as an environmentally benign and abundant binder shows improved bonding and helps to improve the dispersion of the active mass. It also may cause an improvement of the redox reversibility [160] and the rate capability [161]. Other binders, such as polyvinylpyrrolidone (PVP)/polyethyleneimine (PEI) show similar abilities [162]. Furthermore, the water-soluble binder SBR/CMC (styrene-butadiene rubber/carboxyl methyl cellulose) favors a uniform distribution and a network-like cathode structure [163].

Porous carbon structures: A straightforward approach to achieve favorable conductivities is to mix the insulating active material with porous carbons. Depending on the major pore size, *d*, they are distinguished as microporous carbon (*d* < 2 nm), mesoporous carbon (2 nm < *d* < 50 nm) or macroporous carbon (*d* > 50 nm). Especially microporous carbons combine electronic conductivity with an ability to trap polysulfides as first published by Wang et al. in 2002 [164]. Zhang et al. claimed that micropores can work as micro-reactors confining the active mass in the cathode [165]. In more recent studies by Guo and coworkers, an effective steering of the chain length of the active material was obtained by pore sizes smaller than S_8_ molecules of orthorhombic sulfur needing a space of about 0.7 nm [166–168]. The shorter chain length polysulfides show strong adsorption to the carbon matrix and the unfavorable transition between S_8_ and 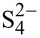 with intermediate polysulfides is hindered, resulting in high cycle life at a lower discharge plateau of 1.9 V [153,169].

**Figure 20:**
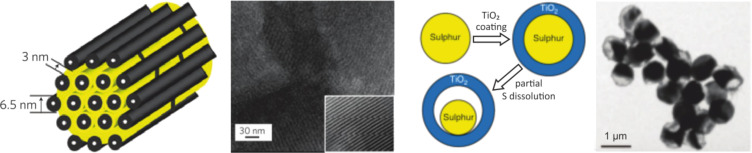
Schematic diagram of the interconnected pore structure of mesoporous CMK-3 impregnated with sulfur (left). TEM image of the impregnated and the pristine (small inset) CMK-3. Figure adapted with permission from [178], copyright 2009 Macmillan Publishers Ltd. Process of formation of S–TiO_2_ yolk–shell structures via core–shell formation and partial dissolution of sulfur (right) [180]. TEM image of the yolk–shell structure with nanoparticles of 800 nm size and shell thickness of 15 nm. Figure adapted with permission from [180], copyright 2013 Macmillan Publishers Ltd.

Especially for microporous supports, a sulfur loading exceeding 50% is difficult due to the limited overall porosity that is provided by microporous carbons [165,169–171]. Also mesoporous carbons are able to trap polysulfides and provide space for a higher sulfur loading [127,170]. As published by Li et al. [172], there is always a tradeoff between complete filling with sulfur resulting in topmost energy density and partial filling leading to better battery performance but lowering energy output. Macroporous supports have been less investigated despite of their high pore volume, as the open structure does not seem to favor polysulfide confinement. However, when immobilizing the polysulfides by providing strong interaction to the matrix [170,173–174] or the use of a highly viscous electrolyte [175], macroporous carbon frameworks may be useful. For both meso- and macro-porous supports, nitrogen doping is promising to improve polysulfide confinement [176]. Bimodal or hierarchical porous carbons were used as compromise to combine confinement of sulfur in small pores while enabling also a higher sulfur loading due to larger pores. Bimodal pore structures were first published by Liang et al. [177]. Although possessing a 3D structure (see below), it should be noted that the CMK-3 ordered mesoporous carbon published by Ji, Lee and Nazar [178–179] was a major starting point for studying tailored, hierarchical carbon materials (see Figure 20).

A range of other special carbon nanostructures have been tested for Li/S_8_ batteries. They are applied in pure form or in combination with conventional carbon materials such as carbon black or activated carbon. Interwoven networks can be obtained by using carbon fibers or nanotubes, for example [179,181]. Cao et al., Zhou et al. and others have reported on sandwich-like electrodes with two graphene layers incorporating the active material, one used as a lightweight current collector, the second used as a barrier for polysulfides [33,182–183]. On the other hand, graphene oxide sheets have been used for wrapping poly(ethylene glycol) covered sulfur particles to obtain confining structures [184].

To completely avoid polysulfide leakage, core–shell- or yolk–shell-structures have been developed to confine the active material inside their electronic and ionic conductive hull. Hollow carbon spheres (void up to 500 nm) with porous shell (up to 50 nm thickness) can be obtained via a hard template nanocasting [157], for example. However, when dealing with an active material that undergoes volumetric expansion and constriction during cycling, closed structures can break. Therefore “yolk–shell”-structures have been suggested that leave enough room for expansion. The latter approach was published by Cui and coworkers [180] comprising sulfur nanoparticles as yolk inside a TiO_2_ shell. The material showed excellent stability for more than 1000 cycles and high Coulombic efficiencies, but only low cathode loadings were reported.

Binder-free electrodes: As the additional weight of the binder reduces the overall energy density of Li/S_8_ cells, binder-free electrodes are studied as alternative. The preparation of binder-free electrodes also avoids the use of often toxic solvents that are necessary for conventional electrode preparation. Elazari et al. reported on a carbon fiber cloth that was able to maintain mechanical strength and conductivity during cycling [170], for example. Vertically aligned carbon nanotubes (VACNTs), directly grown via CVD-process on a metal current collector were published by Dörfler et al. [185]. The high void volume inside the ≈200 µm thick (94 vol %) films was especially favorable for high sulfur uptake, which was later on shown by Hagen and Dörfler et al. [185–186]. Vertically aligned CNTs without a substrate were produced by Zhou [187] using an aluminum anodic oxidized template. Another attempt was published by Manthiram et al., using self-interweaving MWCNTs as free-standing electrodes [188]. Overall, binder-free electrodes might be a viable alternative to standard electrodes. Areal loadings of 7.1 mg/cm^2^ yielding areal capacities of about 5.5 mAh/cm^2^ (50% S utilization) were achieved, although at a low rate of 5/C, for example [185]. Lower loadings allow higher rates of up to 3.5C with specific capacities around 700 mAh/g after 25 cycles [187]. However, reports of more than 100 cycles have not been published yet.

Lithium–sulfide cathode*:* Li/S_8_ cells are usually assembled in the charged state which is less ideal considering safety. Cell assembly in the discharged state, that is, with Li_2_S as positive electrode is intrinsically more safe and has another advantage: The use of anode materials such as Si [189] and Sn [190] and other alloys becomes feasible [189,191–192]. Beginning in the 1970s [193], numerous approaches for Li_2_S cathode formation and investigation on the basic principles have been published. As claimed by Yang et al. [194], when cycling Li_2_S as a cathode material, the first charge is hindered by a potential barrier originating from the slow charge transfer during the oxidation of Li_2_S to Li_2−_*_x_*S, requiring a higher cut-off voltage up to 4 V. Beyond, the hygroscopic property of Li_2_S prohibits handling in air. As stated above, Li_2_S is also an ionic and electronic insulator and requires conductive agents to function as an electrode, hence, comparable approaches to the S composite cathodes have been used [189–190,192,195]. More interesting is the direct chemical synthesis of Li_2_S electrodes without Li_2_S as the starting material: It can be obtained by lithiating a sulfur–carbon composite with stabilized lithium metal powder in situ by compression [196] or with *n*-butyllithium [189]. Archer and coworkers have investigated two different novel approaches towards Li_2_S–C composites: (1) The well-known Leblanc process can be used to reduce sulfates with carbon [197] and (2) Li_2_S builds strong crosslinks with the nitrile groups of polyacrylonitrile (PAN) [198]. Both result in Li_2_S–C composites after carbonization and show promising results. Recently, Lin and coworkers used the reaction of Li_2_S and P_2_S_5_ in THF to form a Li_2_S–Li_3_PS_4_ core–shell structure [199].

3.2.1.2 Electrolytes: The electrolyte will probably play the most fundamental role in the Li/S_8_ battery – potentially even more important than the cathode microstructure, as the solubility of polysulfides and hence the shuttle-effect are dramatically affected by the solvent [121,200–202]. Furthermore, the electrolyte has to be suitable for both the highly reactive Li anode and the sulfur-composite cathode with its special requirements. One important property is good polysulfide solubility to ensure fast and complete reactions between Li and the sulfur [155,200]. On the other hand, a high solubility will accelerate shuttling and loss of active material. Most ether-based solvents can dissolve polysulfides very well, most prominent examples are 1,3-dioxolane (DOL) and 1,2-dimethoxyethane (DME), tetraethylene glycol dimethyl ether (TEGDME, tetraglyme) and sometimes ethers with longer chain length [200,203–205]. Carbonate-based solvents used for conventional LIBs will most likely not be used in future Li/S_8_ batteries. This is due to their reactivity with polysulfides and because they are less compatible with lithium [205–208]. Nowadays, the most common solvent is a binary mixture of a cyclic ether (DOL) and a linear ether (DME), which was found to provide a good overall compromise between sulfur utilization, rate capability, temperature window and anode compatibility [209]. Lithium bis(trifluoromethanesulfonyl)imide (LiN(SO_2_CF_3_)_2_, LiTFSI) is commonly used as a conductive salt. Aurbach et al. pointed out the significance of LiNO_3_ (lithium nitrate) as an electrolyte additive [205,210–215] to build up a both relatively stable and flexible SEI on the lithium anode that suppresses the polysulfide shuttle. However, LiNO_3_ is progressively consumed during cycling and decomposes at the cathode at potentials below 1.6 V [215]. Increasing the conductive salt concentration might alleviate the polysulfide shuttle due to increased viscosity and salting-out effects as stated by Suo et al. [216]. In their work on “solvent-in-salt” electrolytes, an electrolyte with 7 M LiTFSI was found to suppress both polysulfide dissolution and dendrite growth. On the other hand, an increased viscosity generally opposes fast kinetics. Recently, Cuisinier et al. reported on a new “binary” electrolyte comprising a solvent–salt complex (acetonitrile(CAN)_2_–LiTFSI) and hydrofluoroether (HFE) that provide minimum solubility of polysulfides [217]. Hence, a different electrochemical behavior occurs, still forming polysulfide intermediates but suppressing parasitic disproportionation, enabling an earlier Li_2_S formation. Based on the weak Lewis acidity or basicity of ionic liquids (ILs) the solubility of PS is limited as well [218]. Drawbacks of ILs are their high viscosity and therefore lower conductivity resulting in low active mass utilization. The combination with lower viscosity solvents such as DME should be favorable [219] but at the cost of increased polysulfide dissolution. Beyond liquid electrolytes, polymer electrolytes are also used in Li/S_8_ cells that show favorable properties with respect to polysulfide blocking but yet suffer from low ionic conductivity [140,191,213,220]. Despite intense research efforts, the ideal electrolyte has not been identified yet. The possible cure could be to combine a fast conducting liquid electrolyte with a solid lithium-ion-selective separator or solid electrolyte membrane separating both electrodes, thus relying on reliably protected lithium anodes (PLAs) [221–222].

3.2.1.3 Anodes: As the reduction of sulfur occurs at potentials below 2.5 V vs Li/Li^+^, lithium metal is the preferred choice as negative electrode in order to achieve reasonable cell voltages. Moreover, the high theoretical capacity of lithium (3860 mAh/g) is a good match with the high capacity of sulfur (1672 mAhg^−1^). The well-known drawbacks of lithium electrodes (chemical reactivity and dendrite formation) are tried to be minimized by an ex situ applied protection layer or the in situ formed solid electrolyte interphase (SEI) as noted in the previous section. Both in situ and ex situ have to accommodate the changes in volume and morphology during cycling without fracture [223]. To obtain artificial protection layers (artificial SEI), polymer films [224] and inorganic solid electrolytes [221–222] have been applied on the Lithium metal surface. More common is the use of electrolyte additives to favor the formation of a stable SEI, as first published by Aurbach et al. [210,225] referring to LiNO_3_. More recently P_2_S_5_ was suggested as promising additive: A passivating layer mainly consisting of Li_3_PS_4_ with rather high ionic conductivity is formed throughout the reaction of P_2_S_5_ with Li_2_S*_x_* [226]. The SEI formation in situ results from the reaction of lithium with the electrolyte components. Therefore, a fraction of the anode material is irreversibly lost and has to be provided as excess. An alternative route to suppress dendrite growth was suggested by Ding et al. [227]. Here, selected cations (Cs^+^ and Rb^+^) are added that shield emerging lithium dendrites from further Li^+^ access, thus enabling a smoother lithium deposition.

The interest in non-lithium anodes such as Si [189] and Sn [190] has been growing, but these – apart of being pre-lithiated [228] – can only be combined with Li_2_S composite cathodes. Due to the severe volumetric expansion exceeding 300% from Si to Li_15_Si_4_, Si in anodes can only provide stable cycling behavior when being nanosized [229]. Beyond, the theoretical energy density of Li–Si/S_8_ cells is reduced to 1862.45 Wh/kg (3299.25 Wh/L) and to 922.84 Wh/kg (2628.19 Wh/L) for Li–Sn/S_8_ cells, respectively, due to the additional weight and the reduced cell voltage. Also high capacity carbon materials have been studied [230]. The supposed advantages of these anode materials over lithium are improved safety and possibly increased cycle life. But whether this can outweigh the lower energy densities and the disadvantages arising from the decreased cell voltage remains to be clarified.

3.2.1.4 Analytics: Despite the fact that the Li/S_8_ cell has been investigated for a long time, a complete understanding of the redox chemistry and all the electrochemical and chemical processes has still not been achieved. This is foremost due to two reasons: (a) In contrast to the rocking chair LIB, the cell chemistry of Li/S_8_ cells is very complicated, and the reduction of the S_8_ molecule to Li_2_S requires the transfer of 16 electrons. (b) As the processes are particularly sensitive to – for example – the electrolyte composition, often different studies are hardly comparable [124,231–232]. Only recently, in situ methods have been applied to achieve a more realistic overview on the real cell reactions.

**Figure 21:**
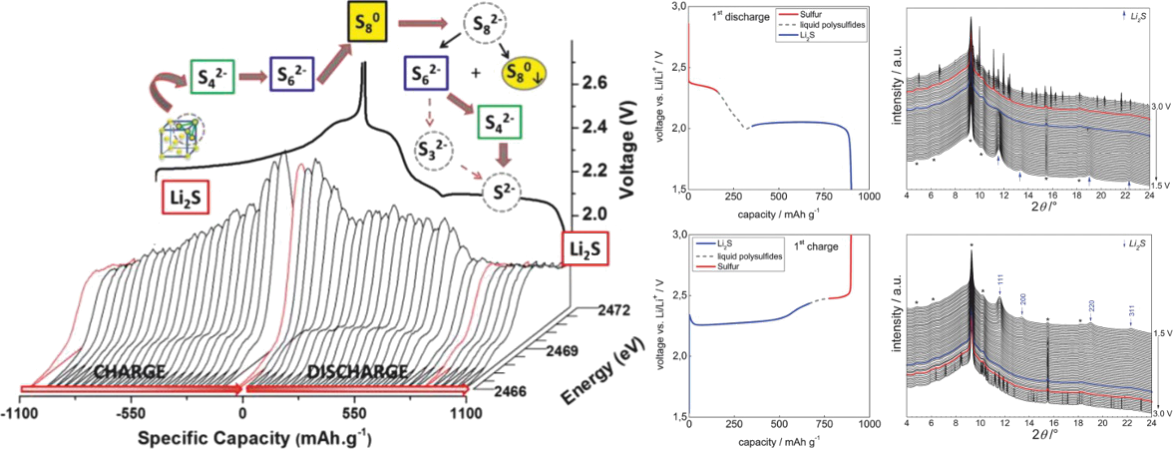
Operando X-ray absorption near-edge spectroscopy (XANES) measurements (left) during first charge and second discharge at 10C rate. The sulfur species denoted on top represent the proposed charge and discharge mechanism based on the measurements. Figure adapted with permission from [241], copyright 2013 American Chemical Society. In situ XRD patterns (right) during discharge and charge at 20C rate with corresponding voltage profiles (left). The * indicates packaging contribution to the patterns, red and blue lines indicate crystalline phases of sulfur and Li_2_S, respectively. Figure adapted with permission from [127], copyright 2013 The Royal Society of Chemistry.

X-ray diffraction is generally a powerful tool to analyze cell reactions in situ [127,233–234] and has been applied to follow the crystalline solid phases appearing during cell cycling. Unfortunately, some discrepancies still remain: The final discharge product Li_2_S is not detected (to be crystalline) in some works ex situ [235] and in situ [233] while others show evidence ex situ [236] and in situ [127,234]. Furthermore, the re-oxidation to orthorhombic sulfur is detected by some groups [233] via XRD, while others see evidence for a different allotrope [127,234] or contradict the formation of elemental sulfur from polysulfides [236–239] at all. One of the most recent studies of in situ XRD is shown in Figure 21 (right), detecting formation of crystalline Li_2_S at the beginning of the 2nd discharge step and precipitation of monoclinic β-sulfur at the end of the charge step. Other methods are necessary to study the soluble polysulfide intermediates. Barchasz et al. proposed a possible mechanism for sulfur reduction in Li/S_8_ batteries by combining high performance liquid chromatography (HPLC), UV–vis absorption and electron spin resonance (ESR) [232]. Further UV–vis analysis was carried out by Patel et al. [240]. Cuisinier et al. published a study on sulfur speciation during cycling using K-edge XANES (X-ray absorption near-edge spectroscopy) [241]. They analyzed intermediate species and followed dissolution and precipitation of redox end members during cycling, finally proposing a cell reaction as denoted in Figure 21 (left). Combination of in situ and in operando techniques is a powerful tool to obtain a clearer qualitative understanding of the cell chemistry. However, challenges remain because – as stated before – the redox chemistry highly depends on the electrolyte, making different approaches hardly comparable. To understand the cell chemistry from a theoretical point of view, microkinetic models of the processes with special focus on the polysulfide shuttling were published by Mikhaylik et al. [123] and Kumaresan et al. [126]. Fronczek et al. used a modeling framework based on computational fluid dynamics (CFD) to develop a one-dimensional continuum model of a Li/S_8_ cell with parameters based on this reference [126] to simulate concentration profiles, voltage and current curves as well as impedance behavior during cycling [231]. Kinetics play a particular role in the Li/S_8_ battery especially because of the divided appearance of fast reactions in solution and sluggish solid state reactions as shown by transient galvanostatic intermittent titration technique (GITT) studies [124]. Hence, both cycling characteristics and performance are affected by the cycling rate and temperature.

3.2.1.5 Alternative cell concepts: As the cell chemistry of Li/S_8_ cells is very different from conventional LIBs, it is also worth considering alternative cell concepts. Negative effects arising from the shuttle effect can be obviated by separating both electrodes with an additional membrane that conducts lithium ions only. This way, polysulfides cannot reach the lithium electrode as suggested by Visco et al, for example [242]. A range of different membranes has recently been tested: lithium ion-exchanged Nafion [243], Nafion-coated polymeric separator [244], Al_2_O_3_-coated [244] and V_2_O_5_-coated [245] separators, and a commercial glass ceramic from Ohara Inc. [185,246]. Manthiram et al. introduced different electronically conductive interlayers between cathode and separator to absorb and reactivate dissolved polysulfides [152]. Obviously, the extra weight and extra resistance of a membrane or layer decreases energy density and rate capability, respectively. However, with the current state-of-the-art, it might be the only reliable cure to the shuttle effect apart from designing an all solid state sulfur battery. This latter attempt may imply new challenges, including (1) low ionic conductivity of solid electrolytes compared to liquid electrolytes for most solid Li-ion conductors, (2) stability of the interface SE/Li-anode and (3) sluggish interfacial kinetics at both electrodes. Additionally, as the ionic contact of the active mass is no longer provided by the liquid electrolyte, a reasonable fraction of finely dispersed ion conductor has to be introduced into the cathode architecture. This leads to a further decrease in energy density. However, with solid electrolytes approaching conductivities that are on par with liquid electrolytes, that is, members of the thio-LISICON (Li Super Ionic Conductor) and Li_2_S–P_2_S_5_ families [247–252], all-solid-state lithium–sulfur batteries might be an attractive option. Moreover, avoiding flammable liquid electrolytes would be an important advantage with respect to battery safety.

**Figure 22:**
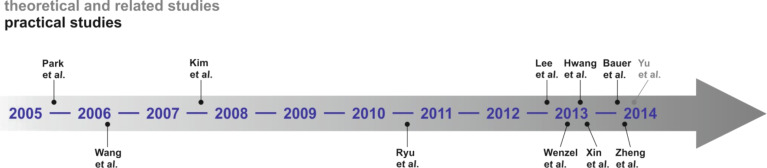
Literature timeline of research papers on room temperature Na/S_8_ batteries (ranked after date of acceptance). Experimental studies: all journal publications in which full discharge–charge capacity profiles were shown for at least one complete cycle. The paper by Yu et al. [253] describes a related concept based on a catholyte*.*

**3.2.2 The sodium–sulfur (Na/S_8_) battery:** The large amount of research publications on lithium–sulfur batteries is in stark contrast to what has been reported on the cell chemistry of the analogue sodium system. Altogether only a few publications on the room temperature cell chemistry of sodium–sulfur batteries are currently available but – similarly to the Na/O_2_ battery – the majority appeared within the last two years. An overview of the available literature is shown in form of a timeline (Figure 22).

Assuming an ideal discharge process, that is, considering thermodynamically stable solids only, sulfur is subsequently reduced to form different polysulfides (Na_2_S*_x_*, *x* = 2, 4, 5) and finally the end product Na_2_S. The theoretical cell potentials of the different steps can be calculated from the corresponding thermodynamic data (no data was found for Na_2_S_5_):

[5]
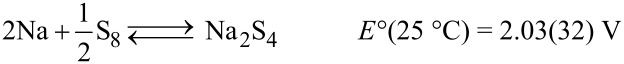


[6]
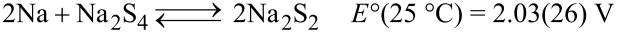


[7]
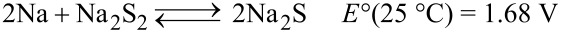


[8]
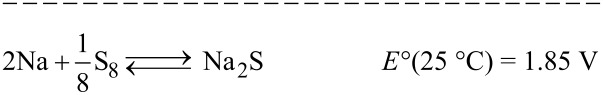


The weighted average voltage of the different steps equals the standard cell potential of the overall reaction. In cells with liquid electrolytes, the reaction path is of course more complex, as, similarly to the Li/S_8_ cell, the phase behavior becomes much more complex as many polysulfides are highly soluble and metastable phases exist. Na_2_S_2_ and Na_2_S, however, are the least soluble compounds in organic solvents so a solid state reaction as stated in Equation 7 is expected at the calculated potential.

Before providing an overview of the current literature it is worth noting beforehand that the overall understanding of the cell chemistry is poor and quite different results have been reported with respect to sulfur utilization and cycle life. This is probably also due to the fact that the experimental conditions were very different (Table 4).

**Table 4:** Cathode- and electrolyte-compositions as well as discharge capacities of all sodium–sulfur cells with an elemental sodium anode that are found in the literature.

Reference	Cathode composition^a^	Electrolyte	1st dis. capacity / mAh/g	10th dis. capacity / mAh/g

Park et al. [254]	70 wt % S 20 wt % C 10 wt % PEO	gel polymer: NaCF_3_SO_3_ in PVDF–TEGDME (1:3:6)	490	105
Wang et al. [255]	70 wt % S/PAN-based comp. (viz. 45 wt % S) 20 wt % CB 10 wt % PTFE	1 M NaClO_4_ in EC:DMC (2:1)	1455	1110
Kim et al. [256]	70 wt % S 20 wt % CB 10 wt % PEO	gel polymer: NaCF_3_SO_3_ in PVDF/HFP–TEGDME (1:3:6)	390	120
Ryu et al. [257]	60 wt % S 20 wt % C 20 wt % PEO	1 M NaCF_3_SO_3_ in TEGDME	540	225
Lee et al. [258]	60 wt % S/HollowC comp. (viz. 27 wt % S) 20 wt % CB 20 wt % PEO	NaCF_3_SO_3_ in TEGDME (4:1 mol %)	1200	600
Wenzel et al. [259]	50 wt % S 40 wt % C 10. wt % PVDF	(a) 1 M NaCF_3_SO_3_ in DME:DOL (1:1); (b) (a) + beta alumina membrane	450 (a) 475 (b)	190 (a) 325 (b)
Hwang et al. [260]	70 wt % S/C–PAN comp. (viz. 32 wt % S) 15 wt % CB 15 wt % PVDF	0.8 M NaClO_4_ in EC:DMC (1:1)	1115	1000
Xin et al. [261]	80 wt % S/(CNT@MPC) comp. (viz. 32 wt % S) 10 wt % CB 10 wt % PVDF	1 M NaClO_4_ in PC:EC (1:1 v/v)	1610	1100
Bauer et al. [262]	42.5 wt % S 42.5 wt % C 12 wt % PVDF 3 wt % PTFE (dry)	(a) 1 M NaClO_4_ in TEGDME, (b) (a) + Nafion coating on PP separator	340 (a) 400 (b)	210 (a) 370 (b)
Zheng et al. [263]	80 wt % HSMC–Cu–S comp. (viz. 50 wt % S) 10 wt % CB 10 wt % CMC (in H_2_O)	1 M NaClO_4_ in EC/DMC (1:1)	1000	690
Yu et al. [253]	60 wt % S 30 wt % CB 10 wt % PVDF	1.5 M NaClO_4_ 0.3 M NaNO_3_ in TEGDME	900	600

^a^PEO: polyethylene oxide, NaCF_3_SO_3_: sodium triflate, PVDF: polyvinylidene fluoride, TEGDME: tri- or tetraglyme, PAN: polyacrylonitrile, NaClO_4_: sodium perchlorate, CB: carbon black, PTFE: polytetrafluoroethylene, HFP: hexafluoropropylene, HollowC: hollow carbon spheres, CNT@MPC: carbon nanotube core@microporous carbon shell particle, PP: polypropylene, HSMC: high surface area mesoporous carbon, CMC: sodium carboxymethyl cellulose.

The first recent report on room temperature sodium–sulfur batteries was published by Park et al. [254] who prepared a cell using a PVDF/tetraglyme-based gel polymer electrolyte with sodium triflate (NaCF_3_SO_3_) as conductive salt (σ = 5.1 ∙ 10^–4^ S/cm at 25 °C). The discharge profile was characterized by two plateaus separated by a sloping potential region, indicative for a stepwise reduction of sulfur over polysulfides. The first discharge capacity was 489 mAh/g and a rapid capacity fading was observed for the subsequent cycles. The authors concluded that a mixture of Na_2_S_2_ and Na_2_S_3_ has been formed during discharge and some sulfur remained inactive. Similar results were obtained for a PEO-based polymer electrolyte but at 90 °C [264]. Later on, the same group (Kim et al. [256]) studied the cell with gel polymer electrolyte in more detail. Again, a similar behavior was found with a capacity of 392 mAh/g for the first discharge followed by a rapid capacity decay. Moreover, the impedance of the cell increased during cell storage which was attributed to the growth of a passivation layer between sodium anode and the gel polymer electrolyte.

Wang et al. [255] reported on a Na/S_8_ cell with liquid electrolyte (NaClO_4_ in EC:DMC) with a high capacity of 1455 mAh/g (or 655 mAh/g_cathode_) and stable cycling over 20 cycles. The cathode material was prepared by heat treating a mixture of PAN and sulfur under inert atmosphere [176]. The sulfur induced the cyclization of the PAN polymer forming H_2_S. The resulting composite consisted of heterocyclic structures and it was suggested that excess sulfur was finely dispersed and eventually covalently bonded to the carbon. The enhanced interaction between sulfur and carbon might explain the high sulfur utilization and stability, at the same time it might be the reason for the unexpected shape of the voltage profile and the lower average cell voltage. No further characterization of the discharge or charge products was provided.

**Figure 23:**
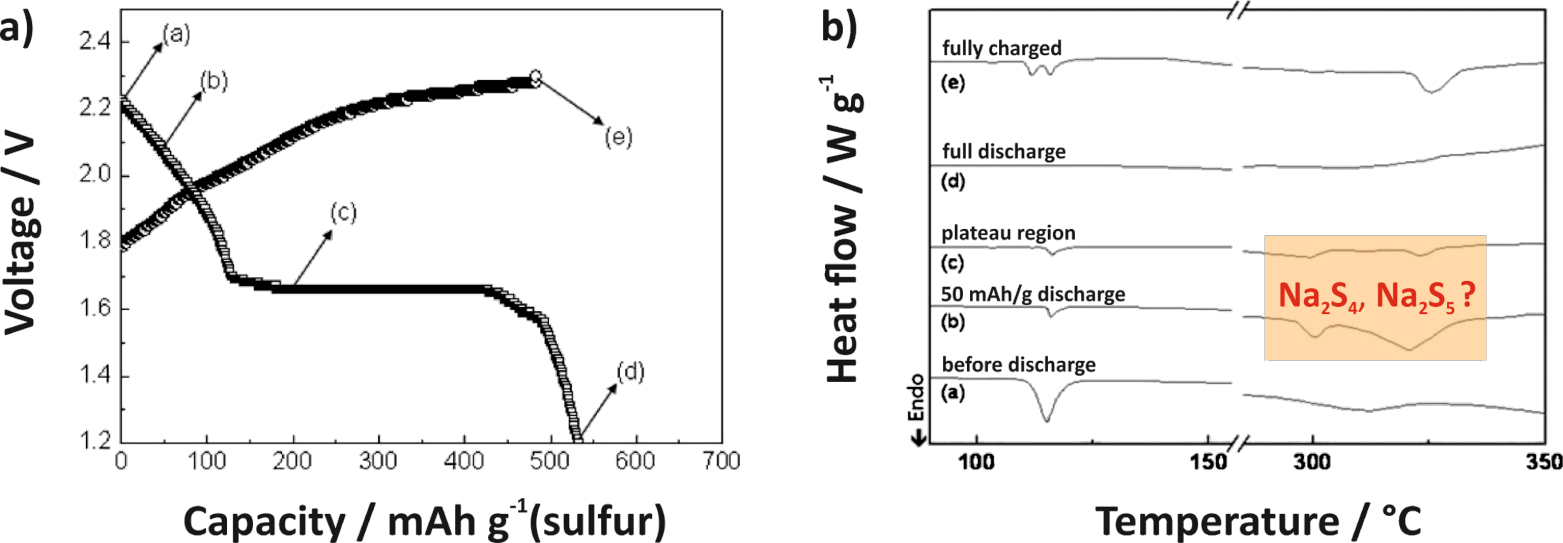
(a) First discharge–charge curve of a Na/S_8_ battery with liquid electrolyte at room temperature and analysis points of sulfur electrode such as (a) pristine, (b) discharged to 50 mAh/g sulfur in sloping region, (c) discharged to 200 mAh/g sulfur in plateau region, (d) fully discharged to 1.2 V and (e) fully charged to 2.3 V. (b) DSC curves of the sulfur electrode with various cut-off voltage conditions as shown in (a). Figure adapted with permission from [257], copyright 2011 Elsevier.

In 2011, Ryu et al. [257] studied the performance of Na/S_8_ cells in a liquid ether based electrolyte (NaCF_3_SO_3_ in tetraglyme). Again, the discharge profile and capacity (538 mAh/g) were comparable to what the same group reported for the cell with gel polymer electrolyte. The voltage profile is shown in Figure 23. In order to provide further insight into the cell reaction, electrodes at different states of discharge and charge (points (a) to (e)) were characterized by differential scanning calorimetry (DSC) (Figure 3b). As several Na_2_S*_x_* polysulfides are thermodynamically stable, their presence in the electrode might be confirmed over their melting points, as shown in the phase diagram in Figure 16b. Notably, this is not possible for the Li/S_8_ cell as Li_2_S is the only stable compound with a defined melting point. The DSC curves indicate that the elemental sulfur disappears during discharge (signal at 114 °C disappears) and sodium polysulfides Na_2_S_4_ and Na_2_S_5_ form (signals 303 °C and 321 °C appear). After full discharge, these polysulfides are absent. After charging, the melting points of sulfur and Na_2_S_5_ reappear. Combined with results from XRD the authors concluded that Na_2_S*_n_* (4 > *n* ≥ 2) forms during discharge and sulfur and Na_2_S*_n_* (5 > *n* ≥ 3) during charge. The ideal discharge product, Na_2_S, was not detected.

**Figure 24:**
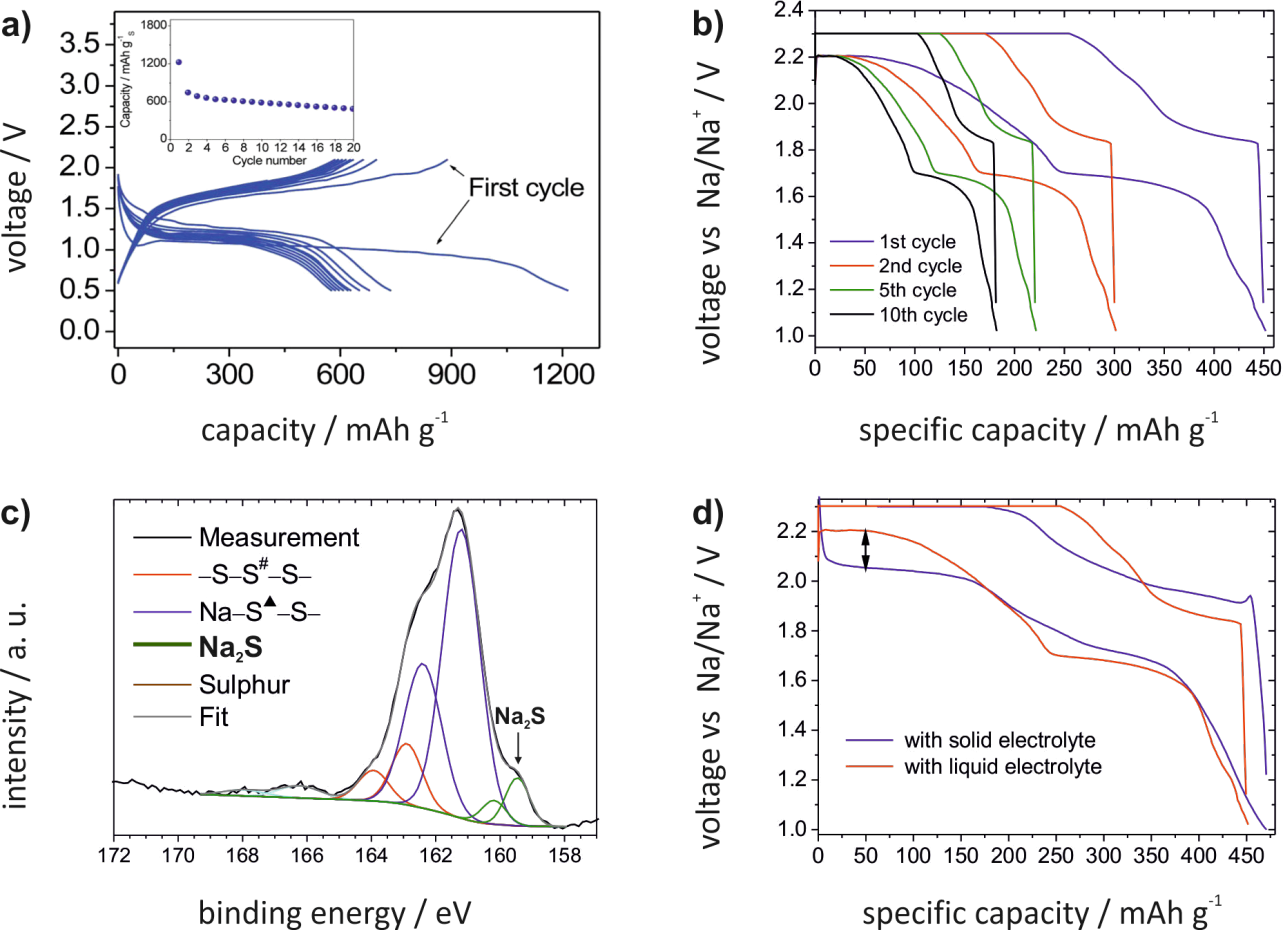
(a) Voltage profiles a of Na/S_8_ cells with a TEGDME-based electrolyte and a nanostructured carbon/sulfur composite as cathode. Figure adapted with permission from [258], copyright 2013 The Royal Society of Chemistry. (b) Voltage profiles of a Na/S_8_ cell with DOL:DME-based electrolyte and a cathode based on commercial materials. (c) XPS spectra of the sulfur electrode after discharge. (d) Comparison ov voltage profiles recorded at a rate of 10C for Na/S_8_ cells with or without solid electrolyte. Figure adapted with permission from [259], copyright 2013 Elsevier.

Lee et al. [258] studied the performance of a sodium–sulfur battery with the same ether-based electrolyte (NaCF_3_SO_3_ in tetraglyme), but using a cathode based on a composite of hollow carbon spheres and sulfur. The cell showed a high initial discharge capacity (1200 mAh/g with a low voltage cut-off at 0.5 V) with the following 20 cycles achieving around 600 mAh/g. At the same time, the discharge potential was only around 1 V (see Figure 24a). No further characterization on the discharge products was provided. In another configuration, the sodium anode was replaced by a Na–Sn–C composite electrode and so presented the first room temperature sodium-ion sulfur battery.

Wenzel et al. [259] studied cells with an ether based electrolyte. Similarly to the results from Ryu et al., an initital discharge capacity of around 450 mAh/g and poor cycle life was found (see Figure 24b). Both the sodium anode and the sulfur cathode were studied by XPS. It was shown for the first time that – although sulfur reduction was incomplete – the ideal discharge product Na_2_S formed during discharge and disappeared during charging (see Figure 24c). At the same time, a large amount of polysulfides and Na_2_S was found on the sodium anode, indicating a very strong shuttle mechanism – in line what can be expected from Li/S_8_ cells. To prevent this shuttle mechanism, an additional inorganic solid electrolyte membrane (beta-alumina) was implemented. With this hybrid electrolyte system, Coulombic efficencies close to 100% were found and somewhat higher capacities could be achieved during cycling. More importantly, cycling at a reasonable rate of 0.1C was still possible meaning that the solid electrolyte did not significantly increase the cell resistance (Figure 24d). This is different from Li/S_8_ cells, where so far only poor kinetics were found for cells with free standing solid electrolyte membranes. We note that the availability of commercially available sodium-ion conducting solid electrolytes with good transport properties in the bulk and through the interfaces with liquid electrolytes offers additional opportunities in designing catholyte based cells. Nevertheless the cells still suffered from strong fading which was finally attributed to the decomposition of the PVDF binder in the presence of polysulfides.

Hwang et al. [260] followed the approach by Wang et al. and produced a composite based on heat treating a mixture of PAN and sulfur; however, PAN nanofibers instead of powder were used. Also here, a carbonate based electrolyte was used (NaClO_4_ in EC:DMC). The cell showed a first discharge capacity of 800 mAh/g and an excellent an cycle life. On the other hand, the sulfur loading was quite small (0.31–0.38 mg/cm^2^). In line with the results by Wang et al., the voltage profile shows an overall sloping behavior and partially low voltages. The authors further showed that the sodium anode was free of sulfur after 500 cycles. This means that polysulfide diffusion from the cathode to the anode can be effectively suppressed by chemically binding sulfur to carbon.

Xin et al. [261] studied the performance of a nanostructured composite consisting of CNTs covered with a microporous layer. The material was designed to alter the reaction mechanism in a beneficial way and had been tested for Li/S_8_ cells by the same group in an earlier study [169]. The idea is that the confinement of nanopores only allows the formation of small compounds, thus, the formation of large S_8_ molecules and large, highly soluble polysulfides is prevented. As a result, the cell reaction is restricted to small S_2−4_ molecules and Li_2_S only, thus improving cycle life and rate capabitlity. The concept also leads to improvements in case of Na/S_8_ cells. An initial discharge capacity of about 1610 mAh/g was found, followed by stable cycling at 1000 mAh/g. Also here, a carbonate-based electrolyte was employed (1 M NaClO_4_ in PC:EC) and the voltage shifts to low values (more than half of the capacity is achieved at voltages below 1.5 V).

Bauer et al. [262] used a polymer membrane to reduce the shuttle mechanism in Na/S_8_ cells with ether-based electrolyte (NaClO_4_ in TEGDME). The membrane was prepared by coating a standard polypropylene separator with Nafion. The initial discharge capacity was around 400 mAh/g, which is similar to what other groups obtained when using ether based electrolytes.

Zheng et al. [263] studied the performance of composite materials containing a high surface area mesoporous carbon, sulfur and copper nanoparticles. The copper nanoparticles were added in order to trap soluble polysulfides by CuS*_x_* formation [263] and a carbonate-based electrolyte was applied (NaClO_4_ in EC:DMC). The first discharge mainly occurs at very low voltage platetau of around 1.0 V and reaches almost 1000 mAh/g. After this activation cycle, stable capacities of around 600 mAh/g are achieved for more than 100 cycles, with Coulombic efficiencies close to 100% and sloping potential curves. Also here, the average voltage values during discharge remain relatively small to what would be ideally expected for the formation of Na_2_S. On the downside, the sulfur loading of the electrode is very small. Although the copper content of the electrodes is small (10%), the cycling behavior shows quite some similarity to a conventional conversion between sodium and CuS*_x_* for which an activation cycle and sloping potentials are well known. Ideally, the conversion reaction of sodium with CuS and Cu_2_S would occur at 1.58 V and 1.39 V, respectively [10].

**Figure 25:**
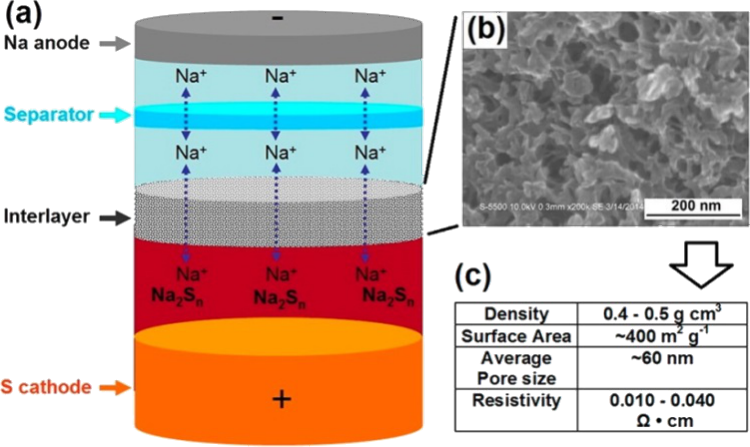
Room temperature sodium–sulfur battery based on shallow cycling between sulfur and soluble long chain polysulfides. An additional interlayer is used to reduce diffusion of polysulfides towards the sodium anode. Figure adapted with permission from [253], copyright 2014 American Chemical Society.

Yu et al. [253] suggested that the often observed capacity fade in Na/S_8_ cells is due to the poor reversibility of the insoluble discharge products Na_2_S*_n_* (1 ≤ *n* < 4). Therefore the group used a cell design optimized for shallow cycling between sulfur and soluble long chain polysulfides with the overall reaction *n*S + 2 Na^+^ + 2 e^–^ = Na_2_S*_n_* (4 ≤ *n* ≤ 8) (see Figure 25). Essentially, this approach is close to a catholyte concept. Evidence for the cell reaction was provided by XPS and UV–vis measurements. A comparable approach was successfully applied in Li/S_8_ cells before by the same group [265–266]. Shuttling of the highly soluble, long polysulfides towards the sodium anode was delayed by implementing an additional nanostructured carbon interlayer (thickness not reported) and using a concentrated electrolyte including NaNO_3_ (1.5 M NaClO_4_ and 0.3 M NaNO_3_ in TEGDME). LiNO_3_ is a well-known anti-shuttling agent in Li/S_8_ cells that protects the lithium anode. Overall, very stable cycling of the cell at 250 mAh/g was achieved for 50 cycles. The average discharge voltage during galvanostatic cycling was around 2.25 V, however, charging curves were not shown so it remains unclear whether the shuttle effect could be prevented.

**Figure 26:**
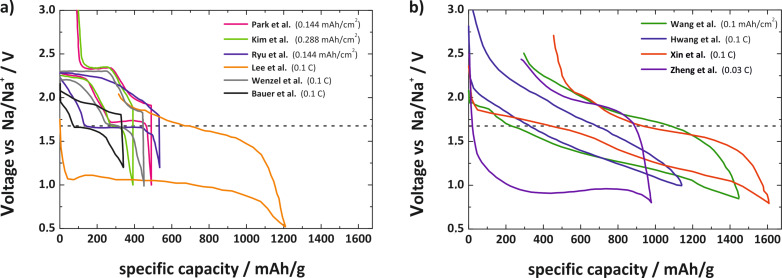
Literature overview on different studies of Na/S_8_ cells with liquid electrolyte operating at room temperature. The comparison shows the first cycle. The data was digitalized from the different publications. (a) Cells with ether-based electrolytes. (b) Cells with carbonate-based electrolytes. The dotted line indicates the lowest cell voltage possible assuming bulk thermodynamics for the solid state reaction from Na_2_S_2_ to Na_2_S. This reaction theoretically accounts for 836 mAh/g.

Overall comparison: In order to compare the different experimental results on Na/S_8_ cells, we digitalized literature data of the first galvanostatic cycle (if available) and plotted them into one diagram (see Figure 26). More data is summarized in Table 4. Obviously some noticeable differences exist.

Results for cells can be grouped according to their voltage profiles as follows:

1. Studies using solvents that are frequently used in Li/S_8_ cells (DOL:DME, tetraglyme) found a discharge behavior that is qualitatively quite similar to what is known from Li/S_8_ cells, that is, one or two plateaus occur at voltages not too far away from the overall expected cell potential (1.85 V). Charging occurs at slightly larger overpotentials compared the Li/S_8_ cell. The main difference, however, is that the achieved capacities are very low. Although it was shown that the theoretical end product Na_2_S forms during discharge, the reaction is incomplete and only about 350–550 mAh/g are found corresponding to an overall composition of Na_2_S*_x_* (3 ≤ *x* ≤ 5). So solvents that work well for Li/S_8_ cells seem to perform bad in Na/S_8_ cells. A notable exception is the work from Lee et al. [258] who used tetraglyme and found a capacity of 1200 mAh/g. But here, discharge mainly occurs at voltages close to 1 V only (cut-off potential of 0.5 V).
2. Studies with carbonate based solvents showed much higher capacities and often also superior cycle life. In one study, the capacity was even close to the theoretical value. At the same time, the voltage profiles of these cells are very different from Li/S_8_ cells and usually exhibit sloping potentials during subsequent cycling and much of the capacity is obtained at voltages below 1.5 V. Such low voltages are also undesired with respect to energy density. One could argue that also the conductive salt might have an influence, however, one can conclude from results obtained for Li/S_8_ cells that this is less likely [205].

Assuming bulk thermodynamics, it is interesting to note that the lowest cell voltage possible is due to the reaction Equation 7:


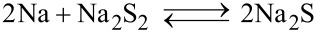


for which *E*° = 1.68 V can be calculated (see above). This reaction contributes to half of the theoretical capacity of sulfur (836 mAh/g). During discharge, the cell voltage should therefore fall below this value at some point which is fulfilled for all results shown in Figure 26. During charge, one should immediately exceed this voltage; however, this is not always the case. It seems that cells with high discharge capacity partially charge below this thermodynamically derived threshold. Assuming that the thermodynamic data is correct, one has to conclude that other reactions take place. In some cases, this unexpected voltage profile might be due to sulfur bound covalently to carbon [255,260] or due to CuS*_x_* [255] formation, however, a clear understanding is missing. Taking these results together, many questions remain and further studies are needed to clarify the link between voltage profile, cycle life, sulfur utilization, and electrolyte composition. As it is well known from Li/S_8_ cells that carbonate based electrolytes are unstable against polysulfides [205,267], future studies should clarify whether side reactions contribute to the high capacities reported for some Na/S_8_ cells.

Moreover, all studies reporting capacities exceeding 1000 mAh/g were only achieved with small sulfur loadings meaning that the sulfur content of the electrode was considerably smaller than 50 wt %. In line with research on Li/S_8_ batteries one has to emphasize the need to increase this value in case an application should become feasible. Given the very early state of research, however, the overall perspective for Na/S_8_ cells is yet unclear, and further work is required to better judge the practical potential.

## Conclusion

Lithium–sulfur and lithium–oxygen cells have attracted enormous interest in the last ten years, and the frequency of publications is still increasing. In the case of Li–sulfur batteries the major challenges have been obvious already at the very beginning (e.g., lithium dendrites, polysulfide shuttle) but still await a proper and effective solution. While incremental improvements can be recognized, it is unclear whether the Li–sulfur battery can finally beat LIB technology with respect to energy capacity. It is interesting to note that the majority of papers deals with the design of carbon/sulfur papers rather than targeting the critical issue of the anode.

In the case of lithium–oxygen batteries the current status is different. After an initial phase of enthusiasm major drawbacks (electrolyte decomposition, carbon instability, the need for pure oxygen) have damped too optimistic expectations, and Li/O_2_ batteries are now again primarily the target of academic research.

As both systems rely on multielectron transfer reactions at the cathode, and as solid phases are being formed and dissolved during cycling, the kinetics are slow compared to LIB and the energy efficiency as also the power density are not competitive yet. This may easily lead to a pessimistic outlook, but this would not be an appropriate conclusion. Rather one should consider lithium–sulfur and lithium–oxygen batteries as attractive targets which have already triggered numerous valuable technical and chemical innovations – but which still require major innovations in electrolyte and electrode design.

In contrast, (room temperature) sodium–sulfur and sodium–oxygen cells have only very recently attracted interest. Obviously, the lower theoretical energy capacity makes sodium-based systems second choice at first glance. On the other hand, sodium systems can provide some specific advantages that might help to overcome the obstacles known from the analogue lithium based cells. Several aspects have been discussed in this review. The availability of Na beta-alumina as highly conductive room temperature solid electrolyte that is also chemically stable in contact with sodium might be an important advantage for designing future cell concepts, for example. Moreover, sodium has the advantage to be much more abundant than lithium.

An intriguing example was also shown for the Na/O_2_ cell, where formation of NaO_2_ as discharge product offers significant advantages compared to the Li/O_2_ cell with respect to energy efficiency and reversibility. Comparing results on metal–oxygen batteries is generally difficult as research groups usually use different cell designs, materials and measurement conditions. However, the shape of voltage profile (voltage hysteresis) gives a first impression on the cell performance with respect to reversibility and efficiency. We therefore suggest using a simple 3 × 3 matrix that allows quick assessment of the overall performance of metal–oxygen cells. The ideal voltage profile corresponds to Type 1A which is not found for any of the metal–oxygen cell yet. Most close to this behavior are Na/O_2_ cells with NaO_2_ as discharge product and are classified as Type 1B. Most other metal–oxygen cells show a Type 2C/3B or Type 3C behavior. Still, very little is known about the cell chemistry of sodium–oxygen cells and it is surprising that different groups find different discharge products. Giving a reasonable explanation for this is an important research task.

Even less is known about the cell chemistry of room-temperature Na/S_8_ cells. The research overview showed that (with one exception), the voltage profile seems to depend on the electrolyte composition. In ether based electrolytes, the voltage profile shows similarity to what is known from Li/S_8_ cells. But although Na_2_S forms as discharge product, only low capacities and poor cycle life is achieved. The situation is different when carbonate based electrolytes are used. Much higher capacities and improved cycle life have been reported. On the other hand, the voltage profiles are much less defined and carbonates might be simply instable against polysulfides as it is known from Li/S_8_ cells. Clearly, there is a need to further understand the cell reaction. At the current state, Na/S_8_ cells are not competitive with Li/S_8_ cells.

In conclusion, given the relatively early state of research, (room temperature) sodium–sulfur and sodium–oxygen cells already show some attractive properties and the recent increase in research activity is a clear sign for the development of two new independent research fields. At the same time, we emphasize that just as for the analogue lithium-based systems, the road towards practical systems is long and might not necessarily lead to application – in particular in view of the energy densities which may finally not beat the LIB. Aiming for low cost stationary energy stores seems most attractive, especially considering the Na/S_8_ system. Progress towards practical devices will be only achieved when challenges of all cell components, that is, anode, cathode and electrolyte, are addressed and side reactions are minimized. Moreover, understanding the of role impurities on the cell reactions need further attention. Innovative approaches in both fundamental research and technical development are therefore needed.
